# MicroRNA and cellular targets profiling reveal miR-217 and miR-576-3p as proviral factors during Oropouche infection

**DOI:** 10.1371/journal.pntd.0006508

**Published:** 2018-05-29

**Authors:** Victor Emmanuel Viana Geddes, Anibal Silva de Oliveira, Amilcar Tanuri, Eurico Arruda, Marcelo Ribeiro-Alves, Renato Santana Aguiar

**Affiliations:** 1 Departamento de Genética, Instituto de Biologia, Universidade Federal do Rio de Janeiro, Rio de Janeiro, Rio de Janeiro, Brazil; 2 Departamento de Biologia Celular e Molecular, Centro de Pesquisa em Virologia, Faculdade de Medicina de Ribeirão Preto, Universidade de São Paulo, Ribeirao Preto, São Paulo, Brazil; 3 Instituto Nacional de Infectologia Evandro Chagas, FIOCRUZ, Rio de Janeiro, Rio de Janeiro, Brazil; University of Texas Medical Branch, UNITED STATES

## Abstract

Oropouche Virus is the etiological agent of an arbovirus febrile disease that affects thousands of people and is widespread throughout Central and South American countries. Although isolated in 1950’s, still there is scarce information regarding the virus biology and its prevalence is likely underestimated. In order to identify and elucidate interactions with host cells factors and increase the understanding about the Oropouche Virus biology, we performed microRNA (miRNA) and target genes screening in human hepatocarcinoma cell line HuH-7. Cellular miRNAs are short non-coding RNAs that regulates gene expression post-transcriptionally and play key roles in several steps of viral infections. The large scale RT-qPCR based screening found 13 differentially expressed miRNAs in Oropouche infected cells. Further validation confirmed that miR-217 and miR-576-3p were 5.5 fold up-regulated at early stages of virus infection (6 hours post-infection). Using bioinformatics and pathway enrichment analysis, we predicted the cellular targets genes for miR-217 and miR-576-3p. Differential expression analysis of RNA from 95 selected targets revealed genes involved in innate immunity modulation, viral release and neurological disorder outcomes. Further analysis revealed the gene of decapping protein 2 (DCP2), a previous known restriction factor for bunyaviruses transcription, as a miR-217 candidate target that is progressively down-regulated during Oropouche infection. Our analysis also showed that activators genes involved in innate immune response through IFN-β pathway, as STING (Stimulator of Interferon Genes) and TRAF3 (TNF-Receptor Associated Factor 3), were down-regulated as the infection progress. Inhibition of miR-217 or miR-576-3p restricts OROV replication, decreasing viral RNA (up to 8.3 fold) and virus titer (3 fold). Finally, we showed that virus escape IFN-β mediated immune response increasing the levels of cellular miR-576-3p resulting in a decreasing of its partners STING and TRAF3. We concluded stating that the present study, the first for a *Peribunyaviridae* member, gives insights in its prospective pathways that could help to understand virus biology, interactions with host cells and pathogenesis, suggesting that the virus escapes the antiviral cellular pathways increasing the expression of cognates miRNAs.

## Author summary

Oropouche Virus causes typical arboviral febrile illness and is widely distributed in tropical region of Americas, mainly Amazon region, associated with cases of encephalitis. 500,000 people are estimated to be infected with Oropouche worldwide and some states in Brazil detected higher number of cases among other arboviruses such as Dengue and Chikungunya. As much as climate change, human migration and vector and host availability might increase the risk of virus transmission. Despite its estimated high prevalence in Central and South America populations, the literature concerning the main aspects of viral biology remain scarce and began to be investigated only in the last two decades. Nonetheless, little is known about virus-host cell interactions and pathogenesis. Virus infection regulates cellular pathways either promoting its replication or escaping from immune response through microRNAs. Knowing which microRNAs and target genes are modulated in infection could give us new insights to understand multiple aspects of infection. Here, we depicted candidate miRNAs, genes and pathways affected by Oropouche Virus infection in hepatocyte cells. We hope this work serve as guideline for prospective studies in order to assess the complexity regarding the orthobunyaviruses infections.

## Introduction

Oropouche Virus (OROV) is the etiological agent of Oropouche fever, an arthropod-borne viral disease characterized by symptoms like fever, headache, myalgia, arthralgia, malaise, photophobia, nausea, vomiting, dizziness, skin rash, and in few cases encephalitis and meningitis [1–7]. It was first described in Trinidad in 1955 [8] and the first Brazilian strain was isolated from a dead pale-throated three-toed sloth (*Bradypus tridactylus*) near a highway construction campsite in Belém, Pará state, northern Brazil [9]. It is estimate that more than 500,000 people were infected in at least 30 outbreaks in South and Central America between 1961 and 2009 [8, 10, 11, 12], placing Oropouche fever as one of the most prevalent arboviral disease in some states of Brazil, after Dengue, Chikungunya and Yellow Fever. However, the virus pathogenesis is still obscure, and Oropouche fever is still considered a neglected disease. During urban outbreaks, the virus is mainly transmitted by its major transmission vector, the midge *Culicoides paraensis* [3, 9, 13]. Other insect species, like mosquitoes of the genus *Aedes* and *Culex*, might also be potential vectors [9]. OROV is classified in the order *Bunyavirales*, *Peribunyaviridae* family, *Orthobunyavirus* genus, as Bunyamwera Virus, La Crosse Virus and the recently discovered Schmallenberg Virus [14]. The order *Bunyavirales* is the largest virus order, containing several viruses implicated in the etiology of relevant human diseases, such as La Crosse Virus (LACV) and Oropouche Virus (*Orthobunyavirus*), Rift Valley Fever Virus (RVFV) (*Phlebovirus*), Crimean-Congo Fever Virus (CCFV) (*Orthonairovirus*) and the rodent-borne Hantaan Virus (HTNV), Andes Virus (ANDV) and Sin Nombre Virus (SNV) (*Orthohantavirus*). OROV has a tri-segmented negative strand RNA genome with a small segment (S) that encodes the nucleocapsid protein N and a non-structural protein NSs; a medium (M) segment that encodes the glycoproteins Gc and Gn and another non-structural protein, NSm, and a large (L) segment that encodes the viral RNA-dependent RNA polymerase (RdRP) [15]. Despite its relevance as a human pathogen and its high prevalence in South America, little is known about OROV replicative cycle, pathogenesis and virus-host interactions. A recent study demonstrated that the OROV entry in HeLa cells is dependent on clathrin-coated pits [16]. Another report showed the relevance of MAVS, IRF-3 and IRF-7, components of the innate immune response, in restricting OROV infection in knockout mice models and non-myeloid cells [17]. Despite that, the virus pathogenesis and the cellular pathways regulated by OROV infection are not known in detail. Gene expression and post-transcriptional regulation is mediated by short non-coding RNAs (microRNAs, miRNAs or miR) that plays important roles during virus replication.

MicroRNAs span between 19–22 nucleotides in length and their first description was made in nematodes [18, 19], though now they have been identified in several phyla of plants and animals [20], and even in viral genomes [21]. In mammals, they can be generated from intronic and exonic regions of protein-coding genes or intergenic regions [22]. They can be found as single miRNA genes or in clusters that encodes long precursor molecules, the pri-miRNA, ranging from hundred to thousand nucleotides in length [23, 24]. Pri-miRNAs begins to be edited in the nucleus by the enzyme Drosha into pre-miRNAs, shorter 70 nucleotides long molecules with hairpin structures [25, 26]. Those pre-miRNAs are exported from the nucleus into the cytoplasm by proteins such as exportin 5 and RAN-GTP [27], and are further processed by Dicer into a 22 nucleotides long double-stranded RNA (commonly referred as miRNA:miRNA*) [28, 29]. The double-stranded RNA is loaded into an Argonaute-driven RNA induced silencing complex (RISC), which selects one strand and binds to a target mRNA (commonly in the 3’-untranslated region, or 3’-UTR region) [30, 31] by base complementarity. The miRNA interaction with its target mRNA induces gene silencing by degradation (when full complementarity between the miRNA and the target sequence occurs) [32], or translational inhibition (in case of partial complementarity) [33, 34]. Since the seed sequence (the minimal complementarity site between miRNA and mRNA) is usually 7–8 nucleotides long, a single miRNA could regulates expression of several genes, as well as a single gene could be regulated by many miRNAs [35, 36].

MiRNAs have already been described influencing disease progression, pathogenicity and replicative cycle of several viruses, being either inhibitory or stimulatory of the infection [37, 38]. The liver-specific miRNA-122 stimulates HCV translation, stabilizing and protecting the 5’-UTR of viral RNAs from degradation, leading to an accumulation of the same in the cytoplasm [39–42]. In resting CD4+ T lymphocytes, HIV-1 viral production is impaired by cellular miRNAs that contribute to establish the viral latency [43]. Another miRNA, miR-29a, targets HIV-1 RNA to accumulate in RNA processing bodies (P-bodies), inhibiting virus infection through translation suppression [44]. Even different strains of the same virus can elicit different miRNA regulation responses, as demonstrated for the highly-pathogenic avian-derived *Influenza* A H7N7 strain and the low-pathogenic swine-derived *Influenza* A H1N1 strain [45], suggesting that miRNA signature profiles could raise clues about pathogenicity variation.

Concerning miRNA regulation by bunyaviruses, a study with pathogenic and non-pathogenic strains of hantaviruses demonstrated the variation on miRNA profile among the different specie-specific viruses and cell types [46]. Another study with the Hantavirus Respiratory Syndrome (HPS)-causing agent, Andes Virus (ANDV), identified down-regulation of miR-126 expression, a miRNA that acts as regulator of SPRED1 [47]. Increased expression of SPRED1 was suggested to be one of the mechanisms that augment endothelial cells permeability, leading to HPS. A recent study with PBMC of patients presenting acute hemorrhagic fever caused by the Crimean-Congo Hemorrhagic Fever Virus (CCHFV) showed the deregulation of several miRNAs, some of them associated with innate immunity and viral escape mechanisms [48]. The only study with phleboviruses described the association between miR-142-3p and the endocytic vesicle protein VAMP3, suggesting a control mechanism for the intracellular trafficking of Uukuniemi Virus (UUKV) [49].

Due to the scarcity of information regarding the regulation of bunyaviruses by miRNA and the increasing necessity of better understanding of virus-host interactions of relevant emerging pathogens, we aimed to evaluate and identify the cellular miRNA profile and target genes induced by OROV infection *in vitro*. We demonstrated that miRNAs miR-217 and miR-576-3p, differentially expressed during infection, could be regulating crucial pathways, like innate immunity response, mainly in upstream proteins of interferon-β induction pathway (adaptor and kinase proteins, as well as transcription factors), protein shutoff and apoptosis.

## Methods

### Cell lines, virus and infection

Cell lines Vero (*ATCC*, *CCL-81*), U87-MG (*ATCC*, *HBT-14*) and HeLa (*ATCC*, *CCL-2*) were maintained in DMEM (*Gibco*) supplemented with 10% v/v Fetal Bovine Serum (FBS) (*Gibco*) and 1% v/v of penicillin-streptomycin (10.000 U/ml-10.000 μg/ml) (*Gibco*) at 37°C and 5% CO_2_. HuH-7 cells were maintained in DMEM without sodium pyruvate (*Gibco*) supplemented with 10% v/v *HyClone* serum (*GE Life Sciences*), 1% v/v antibiotics, 1% 200 mM L-Glutamine (*Gibco*) and 1% v/v non-essential aminoacids (*Gibco*) at 37°C and 5% CO_2_. Jurkat (*ATCC*, *TIB-152*) and THP-1 (*ATCC*, *TIB-202*) were maintained in RPMI-1640 medium (*Gibco*) supplemented with 10% v/v FBS, 1% v/v antibiotics and 1% v/v sodium bicarbonate (*Gibco*) at 37°C and 5% CO_2_. OROV strain BeAn19991 was originally obtained from the Evandro Chagas Institute and propagated by serial passages in Vero cells by routine methods using DMEM. The OROV stock used in the present experiments was propagated in HeLa cells and titrated to 2 x 10^6^ PFU/ml. Infections were performed at MOI 1 during 1 h at 37°C and 5% CO_2_ in medium without FBS, under biosafety level 3 conditions at a BSL-3 laboratory at Universidade Federal do Rio de Janeiro.

### Virus titration

Virus titration was performed by plaque assay in Vero cells plated at 3 x 10^5^ cells/well in 12 well plates 1 day prior to infection. After 1 h incubation with the virus, cells were replenished by DMEM supplemented with 1% v/v FBS, 1% v/v antibiotics and 1% v/v carboxymethyl cellulose (CMC) (*Sigma-Aldrich*), and incubated at 37°C and 5% CO_2_ during 4 days. Cells were fixed with 4% formaldehyde for 20 min at room temperature, washed in Phosphate Buffered Saline (PBS) (*Gibco*) and stained with 20% v/v ethanol-violet crystal solution for 15 min.

### THP-1 PMA treatment

In order to induce monocyte-to-macrophage differentiation, THP-1 cells were stimulated with 100 nM phorbol 12-myristate 13-acetate (PMA) (*Sigma-Aldrich*) in standard RPMI medium for 24 h or 3 days followed by 5 days incubation at RPMI medium without PMA. Fresh RPMI medium was provided to cells after treatment and before infections. THP-1 derived macrophages cells were infected as described above, at 24 h or 8 days post PMA treatment.

### Flow cytometry and immunofluorescence staining

Cells (10^5^ cells/sample) were fixed with 4% paraformaldehyde for 20 min and permeabilized in 1% v/v Triton X-100 PBS solution. Blocking was performed in 5% v/v Donkey Serum (*Sigma-Aldrich)* PBS solution for 1h at 37°C. OROV infected and uninfected cells were incubated with mouse polyclonal anti-OROV antibody at 1:300 dilution in blocking solution at 37°C for 30 min. Cells were then washed thrice in PBS and incubated with 2 μg/ml *Donkey anti-mouse AlexaFluor 488* secondary antibody (*Thermo Fisher Scientific*) at 37°C for 30 min. After incubation with the secondary antibody, cells were washed and resuspended in PBS. Flow cytometry was performed in Accuri C6 cytometer (*BD Biosciences*). At least 10,000 gated events were counted per experimental replica at FITC channel.

### Cell viability assay

HuH-7 were plated at 2 x 10^4^ cells/well density on 96-well plate and incubated at 37°C and 5% CO_2_ for 12 h. After that, cells were infected as described above. Cell viability was evaluated by CellTiter-Blue (*Promega*) according to manufacturer’s instructions. The fluorescence was measured at SpectraMax Paradigm Multi-Mode Detection Platform (*Molecular Devices*).

### RNA isolation and quality assessment

Total cellular RNA for microarray and target mRNA RT-qPCR analysis was isolated using *MirVana kit* (*Thermo Fisher Scientific*) according to manufacturer’s instructions. RNA quantification and quality was assessed by 2100 Bioanalyzer using RNA 6000 Nano kit (*Agilent Technologies*). Only samples with a RNA Integrity Number (RIN) ≥ 9.0 were used for microarray. Extraction of RNA for miRNA validation with specific primers was performed using PureLink RNA Mini Kit (*Thermo Fisher Scientific*) and quantification and integrity were assessed in NanoVue Spectrophotometer (*GE Life Sciences*). All RNAs were treated with DNase (TURBO DNA-free Kit, *Thermo Fisher Scientific*) before RT-qPCR experiments to avoid DNA contamination.

### MiRNA screening

In order to evaluate the expression profile of miRNAs, an array using Taqman chemistry was performed as follows: 12 h after infection, six independent replicas of mock-infected or Oropouche infected (4 x 10^6^ cells/replica at MOI 1) HuH-7 cells were trypsinized (Trypsin 0.25%, *Gibco*) and the total cellular RNA was extracted and quantified as described above. cDNA was generated using TaqMan MicroRNA Reverse Transcription Kit (*Thermo Fisher Scientific*) with 100 ng of RNA per sample according to manufacturer’s instructions. The cDNA was preamplified using Megaplex PreAmp Primers (*Thermo Fisher Scientific*) and Taqman PreAmp Master Mix (*Thermo Fisher Scientific*) as instructed by manufacturer. The qPCR reaction was performed using Taqman OpenArray Human MicroRNA Panels (*Thermo Fisher Scientific*), Taqman OpenArray Real-Time Master Mix and the OpenArray Accufill system OpenArray real-time robotics (*Thermo Fisher Scientific*). This platform is able to quantify 754 human inventoried miRNAs.

### MiRNA array statistical analysis

R statistical language [50] was used for background correction and data exploratory analysis (*Rn* intensity cumulative curve and High Resolution Melting—HRM graphs) for each RT-qPCR reaction. For relative expression quantification, a four parameters sigmoidal curve adjustment was done using the *qpcR* functions in R language [51]. Quantification cycle (Cq) was determined as the relative cycle to second derivative maximum point of adjusted sigmoidal curve (cpD2). The amplification efficiency was determined at the exponential amplification region, at the mean point between relative cycles to the first derivative maximum point and second derivative maximum point of adjusted sigmoidal curve [expR = cpD2-(cpD1-cpD2)], and calculated as the ratio between the expR corresponding cycle fluorescence and the prior cycle fluorescence. For each miRNA, the amplification efficiency was determined as the mean of efficiencies calculated for the corresponding miRNA. Endogenous small-nucleolar RNAs RNU 44, RNU 48 and U6 RNA were candidates for normalization controls selected by the geNorm method [52]. As an alternative normalization method, the normalization factor was calculated by the geometric mean of all miRNA expressed in each sample [53]. For normalized expression comparison between two sample groups, we performed a non-parametric T-test with 1,000 permutations [54]. For three or more groups comparison we used a one-way non-parametric ANOVA with unrestricted permutation (n = 1,000) followed by a non-parametric pairwise T-test mean comparison with permutation (n = 1,000) followed by Bonferroni correction [54]. Results were presented as mean ± S.E.M (standard error mean). Two-tailed p-values in sample groups’ comparison lower or equal to 0.01, 0.05 or 0.1 were considered as highly significant, significant and suggestive, respectively. The relationship between sample profiles was investigated by Bayesian Infinite Mixtures Model cluster analysis [55] and represented by 2D heatmap with dendrograms (bi-cluster). For the purpose of display in the heatmap, k-nearest neighbors method (k = 5) was performed to predict the missing values in uninfected cells for miR-217, miR-26a-2-3p and miR-92a-5p. After imputation of the missing values, a scaled (Z-score) normalization was performed (subtracted miRNA mean divided by miRNA standard deviation).

### Validation of miRNA through primer designed RT-qPCR

Reverse transcription was performed using miRNA 1st-Strand cDNA Synthesis Kit (*Agilent Technologies*) and qPCR reactions were made with High-Specificity miRNA QPCR Core Kit *(Agilent Technologies*) and forward specific primers for each miRNA investigated. Human U6 RNA forward primer (*Agilent Technologies*) was used as normalization control. All the experiments were done in four independent replicas for each time point and sample group. The qPCR reaction was performed in 7500 Real-Time PCR System (*Applied Biosystems*). The cycling parameters were set for standard SYBR Green method according to manufacturer’s instructions as follow: 95°C– 10 min and 95°C– 10 sec, 60°C– 15 sec, 72°C– 20 sec for 40 cycles. The miRNA forward primers sequences are depicted in S1 Table. Statistical analysis was performed using non-parametrical Mann-Whitney tests.

### Target genes bioinformatics prediction

We only consider a putative target for differentially expressed miRNA (miRNA:mRNA interaction) the ones predicted in at least 3 out of 6 public databases as follows: *TargetScan*, (available at http://www.targetscan.org/index.html) *miRTarget2* (available at http://mirdb.org/miRDB/), *PicTar* (available at https://pictar.mdc-berlin.de/), *miRBase* (available at http://www.mirbase.org/), *TarBase* (available at http://carolina.imis.athena-innovation.gr/diana_tools/web/index.php?r=tarbasev8%2Findex) and *miRanda V3*.*3a*. Interaction network tree was designed using Cytoscape v3.2.1 software (Cytoscape Consortium).

### Gene set enrichment analysis (GSEA)

Ontology enrichment analysis [56] was performed for the predicted targets of the differentially expressed miR-217 and miR-576-3p. The ontologies were enriched mainly to biological processes, molecular function, cellular components, and gene interaction/regulation pathways. Only genes predicted in at least 3 out of 6 databases were considered candidate targets. Gene *Entrez* id for the predict ontologies were used in the *Gene Ontology Database* (GO, available at http://www.geneontology.org/), KEGG (available at http://www.genome.jp/kegg/) and REACTOME (available at http://www.reactome.org/PathwayBrowser) for this purpose. Only genes over represented in hypergeometric tests with p-value ≤ 0.001 were considered.

### MicroRNA inhibitors transfection

HuH-7 cells were seeded (10^5^ cells/replica) in triplicate into 24 wells plate overnight. Negative control inhibitor, miR-217 inhibitor and miR-576-3p inhibitor (*Integrated DNA Technologies*) were transfected at a final concentration of 75 nM using 2 μl of Lipofectamine 2000 (*Thermo Fisher Scientific*) per replica. Green fluorescent short RNA siGLO (*Dharmacon*, *GE Life Sciences*) was used to assess transfection efficiency and establish the miRNA inhibitor concentration for inhibition experiments. 3 h post-transfection, cells were infected with OROV at MOI 1 and RNA were extracted for miRNA quantification (6 h post-infection) or target gene and OROV RNA quantification (18 h post-infection) by RT-qPCR. OROV segment S RNA was quantified using primers and probe [57] with Taqman 2x Universal PCR Master Mix (*Thermo Fisher Scientific*) and normalized by GAPDH using PrimeTime primers and probe mix (*Integrated DNA Technologies*).

### Validation of target mRNA through RT-qPCR

Cells were seeded (10^6^ cells/sample) and infected with OROV at MOI 1. The RNA was extracted at 12 h post infection and reverse transcription was performed using High-Capacity cDNA Reverse Transcription Kit (*Thermo Fisher Scientific*) and 1 μg of RNA. Quantitative PCR was done in six replica per condition using 50 ng/well of cDNA on Custom Taqman Array Fast plates (96 well) (*Thermo Fisher Scientific*) using specific primers and probes and Taqman Fast Universal PCR Master Mix (*Thermo Fisher Scientific*) according to manufacturer’s instructions on 7500 Fast Real-Time PCR System (*Thermo Fisher Scientific*). Statistical analysis was done as described for microarray using endogenous 18S, GAPDH, HPRT1 and GUSB as normalization genes. For target kinetics SYBR Green PCR Master Mix (*Applied Biosystems*) and pre-designed PrimeTime primers (*Integrated DNA Technologies*) were used according to manufacturer’s instruction (for primers sequences see S1 Table).

## Results

### OROV infects hepatocytes and blood cell lines *in vitro*

**Fig 1:**
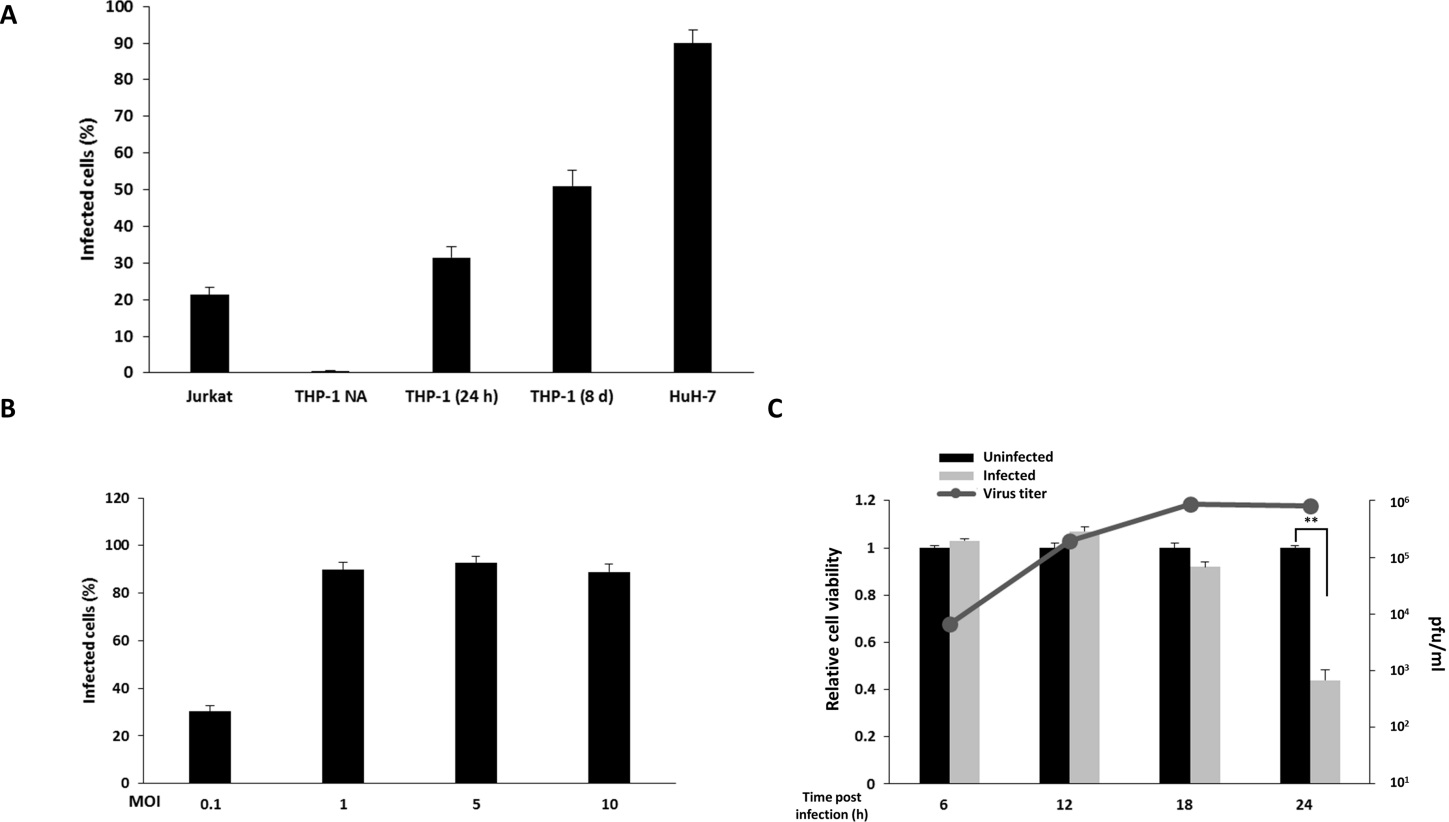
Oropouche infection in blood and hepatocyte cell lines. Jurkat, THP-1 and HuH-7 cells were infected with OROV at MOI 1. At 12 h post infection, infectivity was assessed by flow cytometry using polyclonal anti-OROV antibody. (**A**) Infection in T lymphocyte (Jurkat), monocyte (THP-1) and hepatocyte (HuH-7) cell lines (x-axis) was performed as described in materials and methods section. Error bars represents standard deviation (SD) of four independent experiments. THP-1 NA = THP-1 not activated; THP-1 (24) = activated for 24 h in PMA; THP-1 (8 d) = activated for 3 days in PMA followed by 5 days without PMA. (**B**) OROV infection in Huh-7 cells at different MOIs (x-axis). Infectivity was assessed by flow cytometry. Error bars represents SD. (**C**) Relative cell viability of HuH-7 cells infected with OROV (MOI 1) at different time points. Cell viability was assessed by fluorimetric assay. Absolute values of uninfected cells were set as 1 (left y-axis). Black columns represent uninfected cells and gray columns represent OROV infected cells (MOI 1). Error bars represents SD for five replicates of two independent experiments. Gray line represents mean virus titer in supernatant measured by plaque assay at indicated time points (right y-axis). ** = *p* ≤ 0.01.

In order to expand the knowledge on the range of OROV-permissive cells, blood and hepatocyte cell lines were used to evaluate *in vitro* infection (Fig 1A). T CD4^+^ lymphocytes (Jurkat), monocytes (THP-1) and hepatocytes (HuH-7) cell lineages were infected with OROV at MOI 1 and, at 12 h post infection, infectivity was assessed by immunofluorescence using specific antibodies against OROV proteins and virus-positive cells were counted by flow cytometry. At indicated time points, 21% of Jurkat cells were infected, while THP-1 presented no susceptibility to the OROV infection. THP-1 cells can be induced to differentiate into macrophage by PMA treatment, becoming permissive to some viral infections, as described elsewhere [58–61]. In order to assess if THP-1 cells differentiated into macrophages were permissive to OROV infection, THP-1 cells were treated with PMA for 24 h or for 3 days, followed by incubation in medium without PMA for 5 more days. Differentiation of THP-1 into macrophage-like phenotype was accompanied by microscopy and attachment. At 12 h post infection, 31% and 50% of THP-1 treated with PMA for 24 h or 8 days, respectively, were infected with OROV, suggesting an increasing permissiveness to OROV infection as the cells shift from monocyte to macrophage-like phenotypes (Fig 1A). At the same MOI, HuH-7 cells showed to be more permissive to OROV infection, presenting 90% infected cells at 12 h post-infection (Fig 1A). Based on this result with HuH-7 cells, and considering previous demonstrations that the liver is an important replication site during experimental OROV infection in hamster [62, 63] and mouse [64], we chose the hepatocyte cell line HuH-7 as our *in vitro* model for further experiments.

To assess the most suitable conditions to ensure that most cells would be infected at indicated time points, HuH-7 cells were infected with different MOIs and the infectivity was measured by flow cytometry (Fig 1B). We reached 30% of infectivity at MOI 0.1 with a plateau of 90% in higher concentrations of virus (MOIs 1, 5 and 10), with no further increase of infectivity levels (Fig 1B). To assure that cells were still viable for further experiments, we assessed the cytopathic effect at 6, 12, 18 and 24 h post infection with MOI of 1 using Cell Titer-Blue (Fig 1C). We did not detect cell death associated to the OROV infection at least 18 h post infection. However, only 44% of cells were viable at 24 h post infection. We also quantified the virus titer generated in those cells by plaque assay and at 6 h post-infection, the titer in the supernatant was 6 x 10^3^ PFU/ml (Fig 1C, gray line). As the infection progressed, a peak of 8.6 x 10^5^ PFU/ml could be detected at 18 h post infection, reaching a plateau with no further increase in viral titer at 24 h post infection (Fig 1C, gray line). Based on these data, we proceeded using HuH-7 cells infected with MOI 1 in subsequent experiments to evaluate the virus-host interactions.

### Cellular miRNA signature in HuH-7 cells infected with OROV

**Fig 2:**
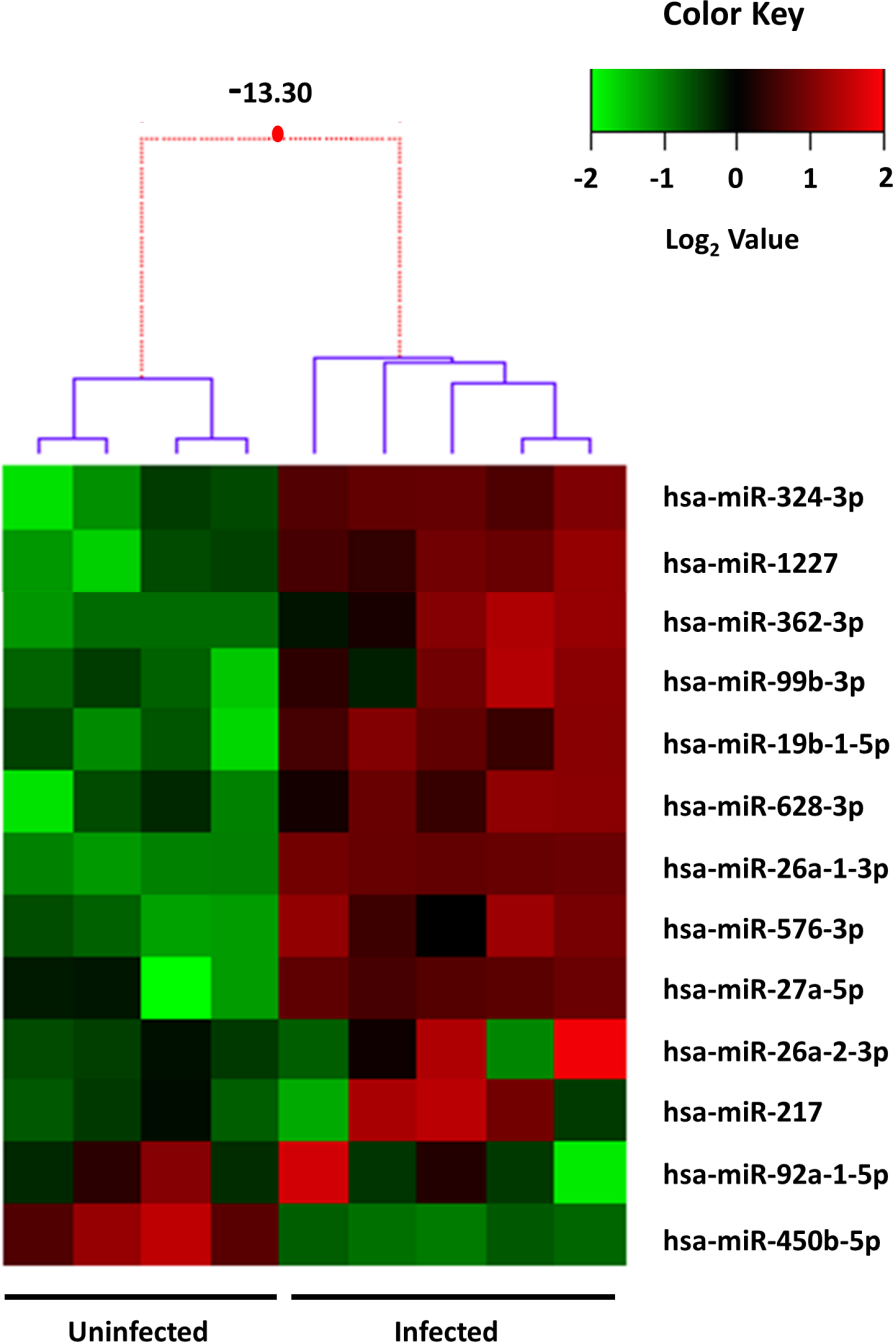
Heat map of differentially expressed miRNAs in OROV infected HuH-7 cells. Depiction of the 13 miRNAs differentially expressed in OROV infected cells relative to uninfected cells. Lines represent individual miRNAs and columns represent independent replicates (four mock infected and five OROV infected). Color scale represents normalized expression levels of miRNAs in the two conditions in log_2_ scale; red denotes up-regulation and green denotes down-regulation, respectively. Dendrogram representing the 1D clusterization of samples and the 2D map corresponding to the levels of standardized gene expression profiles (z-score). Red dotted lines in the dendrogram indicate weak unions, discouraged by the Bayesian clustering analysis. Values represented in the dendrogram branches correspond to log-odds of the union of corresponding branches. For the purpose of display, missing values of uninfected cells for miR-217, miR-26a-2-3p and miR-92a-5p were predicted by k-nearest neighbors method and imputed after normalization as described in *Methods* section.

MiRNAs can be informative of cellular targets modulated by virus infection. In order to identify candidate cellular pathways differentially expressed in OROV infected cells, we performed an exploratory screening of 754 human miRNAs through probe-based RT-qPCR. MiRNAs expression was evaluated in four uninfected (control) and five OROV infected biological replicas at 12 h post infection. We found thirteen miRNAs differentially expressed upon OROV infection in HuH-7 cells with statistical significance: twelve up-regulated after infection and only one down-regulated (miR-450b-5p) (Fig 2 and Table 1). The reproducibility of effects in miRNAs was indicated by small variance noted among biological replicas, as depicted in the heat map hierarchical dendrogram (Fig 2).

**Table 1:** Mean relative expression levels of the 13 differentially expressed miRNAs.

miRNA	Mean Fold Change (Infected/Uninfected)	P-value	Bonferroni Adjusted P-value
**hsa-miR-1227**	**1.95**	**0.0025**	**0.0596**
**hsa-miR-19b-1-5p**	**4.11**	**0.0025**	**0.0596**
**hsa-miR-217**	**NA**	**NA**	**NA**
**hsa-miR-26a-1-3p**	**42.47**	**3.04E-06**	**0.0004**
**hsa-miR-26a-2-3p**	**NA**	**NA**	**NA**
**hsa-miR-27a-5p**	**108**	**0.0329**	**0.1597**
**hsa-miR-324-3p**	**1.73**	**0.0074**	**0.0924**
**hsa-miR-362-3p**	**1.85**	**0.0042**	**0.0653**
**hsa-miR-450b-5p**	**0.215**	**0.0020**	**0.0596**
**hsa-miR-576-3p**	**2.49**	**0.0006**	**0.0377**
**hsa-miR-628-3p**	**2.77**	**0.0062**	**0.0859**
**hsa-miR-92a-1-5p**	**NA**	**NA**	**NA**
**hsa-miR-99b-3p**	**2.21**	**0.0038**	**0.0653**

NA—not applicable since they were detected only in OROV infected cells

The differential expression of the miRNAs in OROV infected cells was classified into three groups: up-regulated, down-regulated and infection-dependent miRNAs (selectively expressed miRNAs). MiRNAs miR-324-3p (1.73x), miR-1227 (1.95x), miR-362-3p (1.85x), miR-99b-3p (2.21x), miR-19b-1-5p (4.11x), miR-628-3p (2.77x), miR-26a-1-3p (42.47x), miR-576-3p (2.49x) and miR-27a-5p (108x) were up-regulated, in OROV-infected cells relative to uninfected cells. MiR-450b-5p was down-regulated 4.65 times in infected cells compared to uninfected cells. The induction of miR-26a-2-3p and miR-217 were inconsistent and observed only in three out of five infected replicas. From the thirteen selected miRNAs from the screening, only miR-576-3p and miR-26a-1-3p sustained significance (p ≤ 0.05, p ≤ 0.01, respectively) after Bonferroni correction according to the method used in this study. Nonetheless, some miRNAs presented borderline limits of significance (p = 0.0595), namely, miR-1227, miR-19b-1-5p and miR-450b-5p (Table 1).

**Fig 3:**
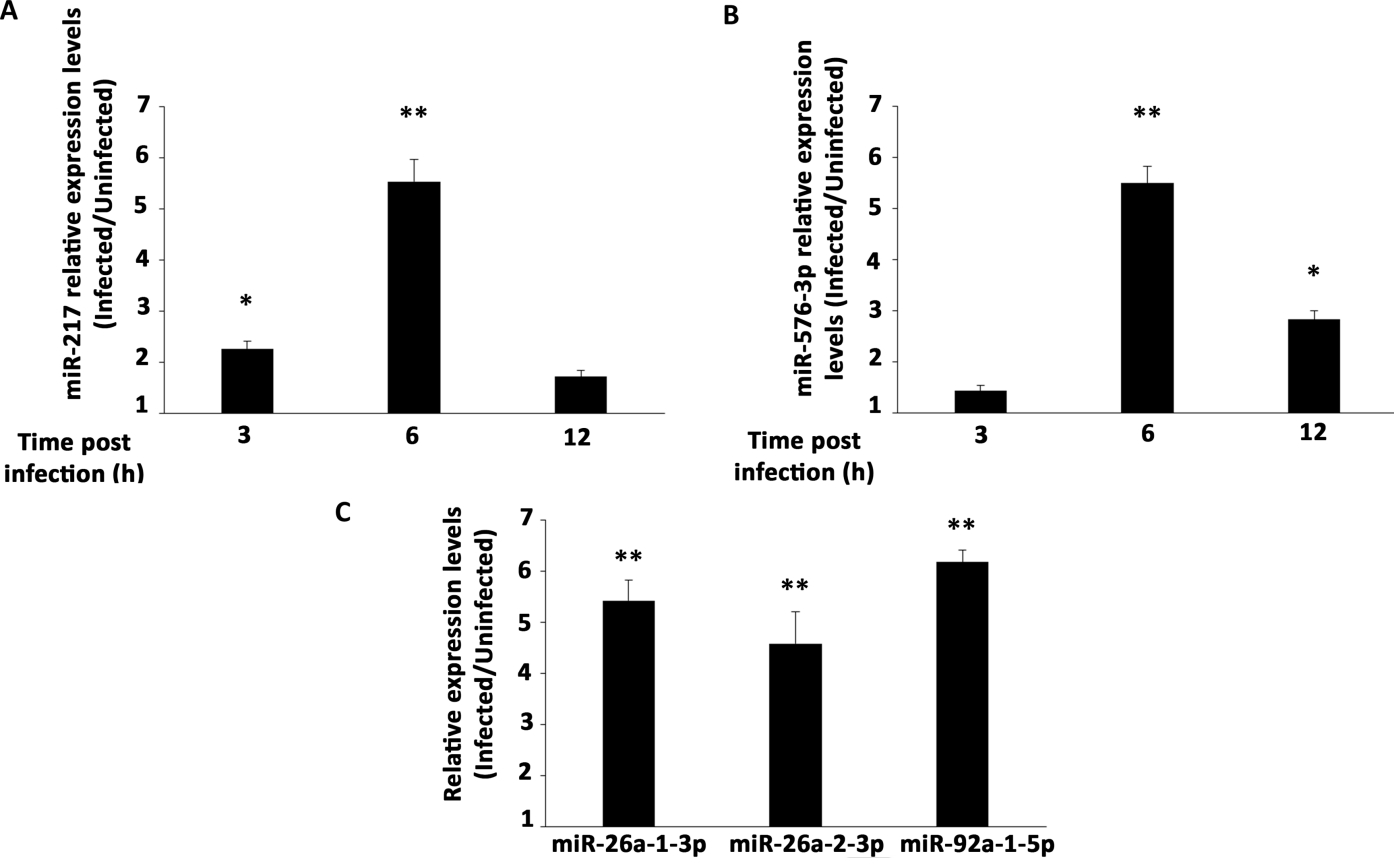
Validation of individual miRNAs expression. RT-qPCR with specific primers for individual miRNAs. Mean fold change of expression levels in OROV infected cells relative to uninfected cells for (**A**) miR-217 and (**B**) miR-576-3p. RT-qPCR was performed at 3, 6, 12 h post-infection. (**C**) RT-qPCR for miR-26a-1-3p, miR-26a-2-3p and miR-92a-1-5p was performed only at 12 h post-infection time point. MicroRNAs expression was normalized by U6 RNA endogenous levels. Error bars represent SD of triplicates of three independent experiments. Asterisks represent significant values compared to non-infected cells. * = p ≤ 0.05; ** = p ≤ 0.01.

In order to validate the miRNAs that were significantly deregulated in the array (miR-26a-1-3p and miR-576-3p) and to verify the expression of the miRNAs only detected in infected cells in the expression profile array (miR-217, miR-26a-2-3p and miR-92a-1-5p), we designed specific primers for each miRNA and checked its expression by RT-qPCR (Fig 3). Our validation experiments showed the same tendency of the miRNAs panel with an increasing expression of both miR-217 (Fig 3A) and miR-576-3p (Fig 3B) during infection, reaching a peak of expression at 6 h post-infection (about 5.5 fold increase for both miRNAs). The kinetics of expression of miR-217 suggests an early induction during infection compared to miR-576-3p, since miR-217 was up-regulated 2.26 times as early as 3 h post-infection while miR-576-3p was only up-regulated 1.44 at the same point. However, at later stages of infection, miR-217 expression was already closer to uninfected expression levels (up-regulated only 1.7 at 12 h post-infection), whereas miR-576-3p was still up-regulated 2.83 times in infected cells, indicating a slightly different kinetics for those miRNAs.

To confirm the robustness of our analysis, we further validated the expression of three other less stable star miRNAs: the highly significant miRNA miR-26a-1-3p and two miRNAs detected only upon infection, miR-26a-2-3p and miR-92a-1-5p (Fig 3C). Those three miRNAs were up-regulated 5.3, 4.5 and 6.3 fold, respectively, at 12 h post-infection in comparison with uninfected cells (p ≤ 0.01). Altogether, these results with specific primers to each miRNA corroborate with our large-scale panel data, identifying miRNAs that are modulated during OROV infection showing the same tendency with different approaches. Star miRNA nomenclature corresponds to passenger strands less favorable to processing by RISC with lower likelihood to regulate gene expression [65, 66]. As most of those miRNAs are previously annotated as star miRNAs (as example of miR-26a-1-3p previously annotated as miR-26a-1*) and some prediction database algorithms use proved interaction as criteria for prediction, we only selected miR-217 and miR-576-3p, both mature strand miRNAs, for further target prediction analysis (one detected only in infected cells and the other one up-regulated significantly upon infection in the array, respectively, and both validated).

### Cellular target genes regulated by miR-217 and miR-576-3p

**Fig 4:**
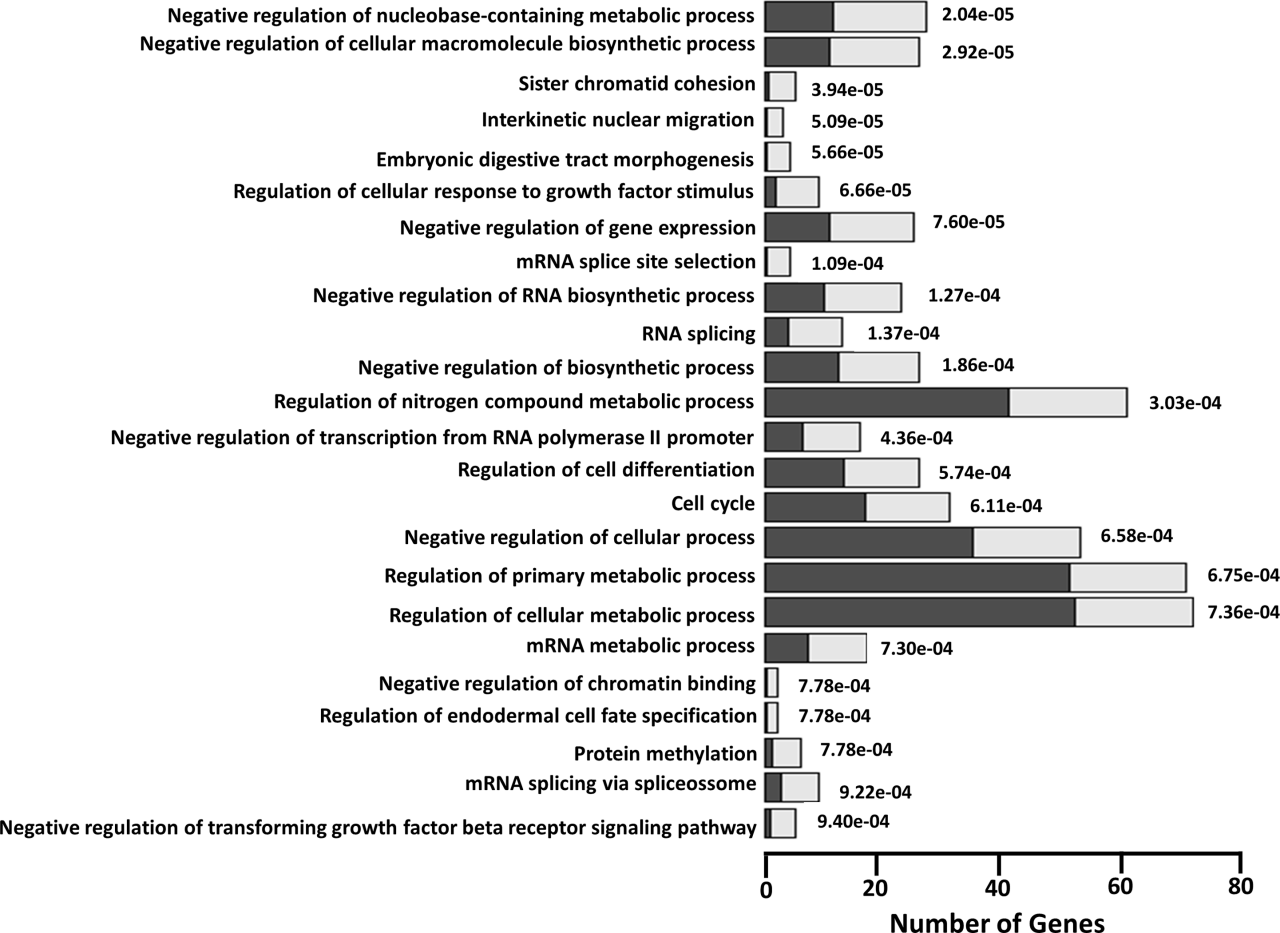
Enrichment pathway analysis of predicted targets for miR-217 and miR-576-3p. Gene set enrichment analysis (GSEA) was performed for all predicted targets using gene ontology available in GO, KEGG and REACTOME databases. Over-represented pathways are depicted in the y-axis. Quantity of genes associated to a specific pathway is represented in the x-axis. Black columns represent the quantity of genes expected to be associated to a determined pathway and gray columns represent the observed quantity of genes associated to the same pathway, respectively. Significance of overlapping is represented by the negative log of P-value for each pathway.

To investigate possible pathways regulated by miR-217 and miR-576-3p during OROV infection, we performed target prediction using TargetScan, miRTarget2, PicTar, miRBase, TarBase and miRanda databases. Target genes predicted by at least 3 out of 6 of those databases were considered candidates. We predicted 195 cellular genes to interact with miR-217, miR-576-3p, or both, using that criterion (S2 Table). We used enrichment analysis with GO, KEGG and REACTOME to identify cellular pathways affected by the predicted targets identified with our selection criteria. Our analysis showed the enrichment of cellular pathways related to regulation of cell metabolic processes, cell cycle and differentiation, chromatin stability and RNA metabolism and expression, suggesting that OROV infection possibly affects cell basic processes and RNA-related regulation processes, as expected for a RNA virus (Fig 4). This can be confirmed by the increasing numbers of observed genes (gray columns) compared with the expected numbers (black columns) for each cellular pathway analyzed (Fig 4). All the analysis showed very significant statistical levels with *p* values < 0,0001.

### Validation of miRNAs predicted target genes modulated by OROV infection

**Fig 5:**
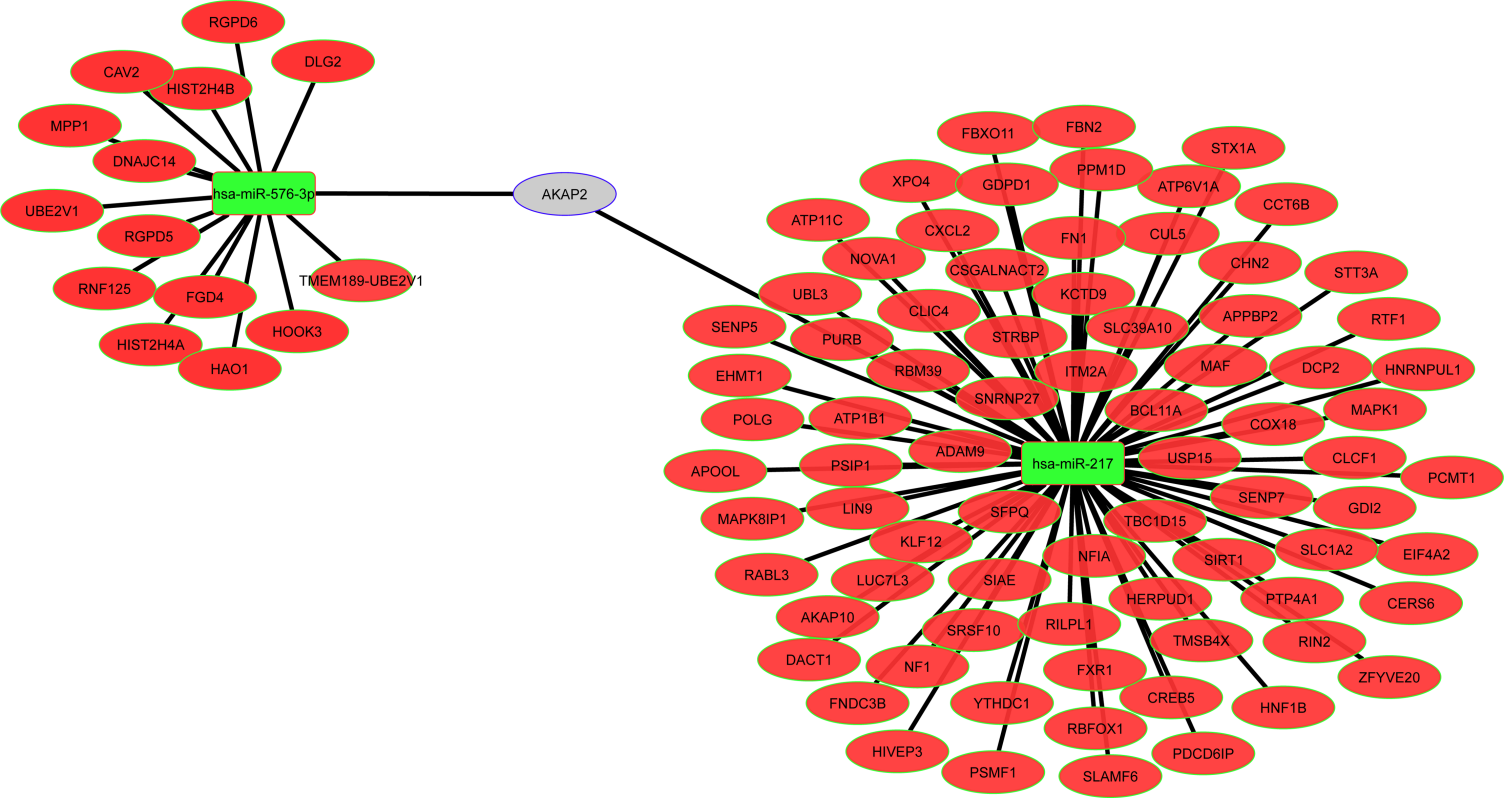
Interaction network between miRNAs and predicted target genes modulated by OROV infection. Network depicting the relation among miR-217, miR-576-3p and 92 selected target mRNAs found in at least 3 out 6 databases. Green rectangles displays the two selected up-regulated miRNAs; red ellipses represent predicted down-regulated mRNAs; gray ellipse represents mRNA predicted to be regulated by both miRNAs.

We selected 95 target genes, which were either in the group of 195 predicted target genes (92 genes) and/or either were already published as target genes for those miRNAs, to evaluate their expression through RT-PCR in OROV infected hepatocyte cells. The selection criterion was based on the function described in the literature or involvement in relevant biological pathways related to RNA viruses such as: intracellular trafficking, apoptosis, innate immunity, gene expression regulation, antiviral restriction factor, protein synthesis regulation and intracellular signaling. The predicted selected targets and their association with miRNAs are depicted in the Fig 5 interaction network (see also S3 Table for a brief description of targets function).

**Fig 6:**
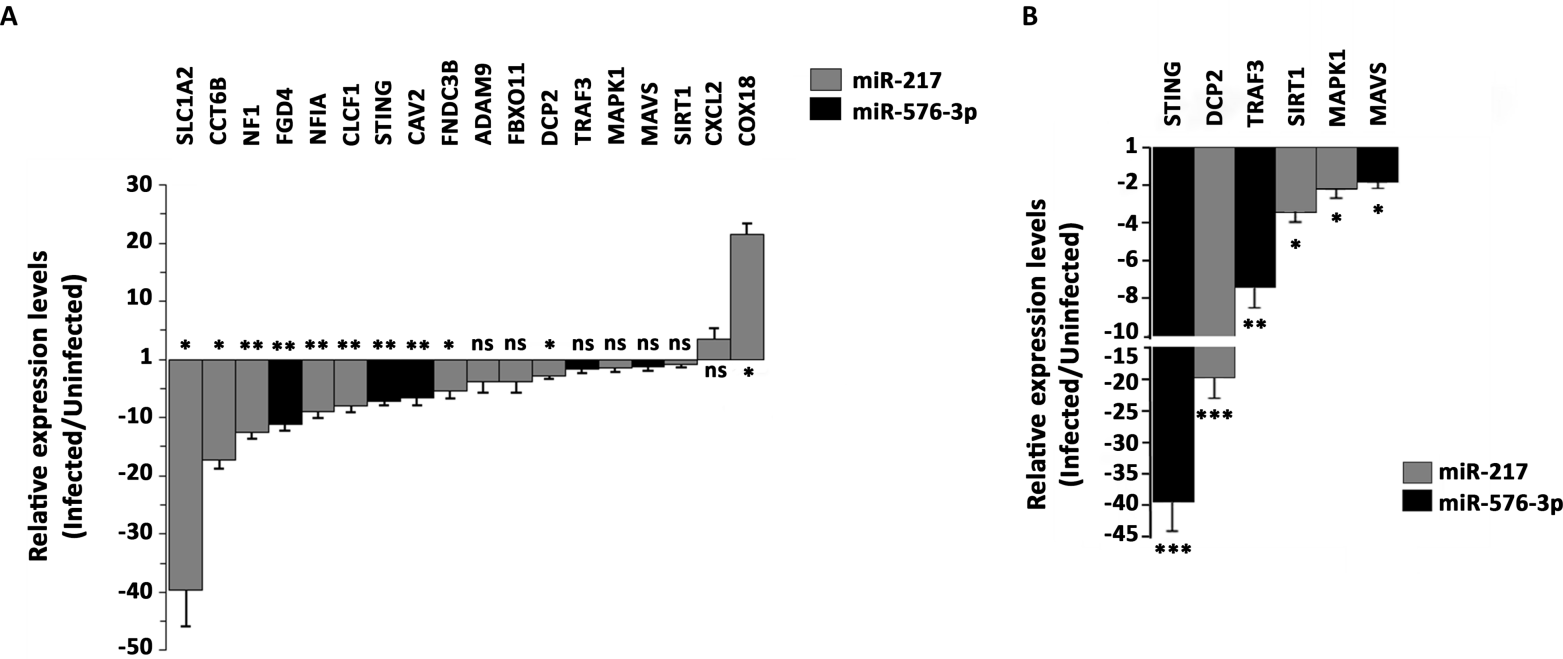
Relative expression levels of selected target mRNAs predicted for miR-217 and miR-576-3p. HuH-7 cells were infected with OROV at MOI 1 and RT-qPCR was done 12 h post infection (**A**) or 24 h post infection (**B**). (**A**) Data denotes mean fold change (y-axis) of infected cells relative to uninfected cells for 18 deregulated mRNA out of 95 selected targets. Gene expression was normalized by endogenous 18S, GAPDH, HPRT1 and GUSB levels. Deregulated target genes are depicted in x-axis. Black columns represent predicted targets for miR-576-3p and gray columns represent predicted targets for miR-217, respectively. Error bars represent Standard Error Mean (SEM) for 6 independent samples. ns = non-significant (0.05 ≤ p ≤ 0.1); * = p ≤ 0.05; ** = p ≤ 0.01. (**B**) Data denotes mean fold change (y-axis) of infected cells relative to uninfected cells for 6 selected targets 24 h post infection. Gene expression was normalized by endogenous GAPDH levels. Black columns represent predicted targets for miR-576-3p and gray columns represent predicted targets for miR-217, respectively. Error bars represent SD for four independent samples. * = p ≤ 0.05; ** = p ≤ 0.01; *** = p ≤ 0.001.

From the 95 selected genes tested by RT-qPCR analysis we showed only the 18 genes that were differentially expressed 12 h post infection in comparison with uninfected cells (Fig 6A). The majority (16 genes) were down-regulated, corroborating with the opposite up-regulation trend of the related miRNAs during infection. The gene expression of membrane anchor protein ADAM9, the component of SCF (SKP1-CUL1-F-box protein) E3 ubiquitin-protein ligase complex F-box Only protein 11 (FBXO11), the TNF Receptor Associated Factor 3 (TRAF3), the Mitogen-Activated Protein Kinase 1 (MAPK1) and the Mitochondrial Antiviral-Signaling protein (MAVS), all had a trending of (0.05 ≤ p ≤ 0.1) down-regulation. They were 3.9, 3.9, 1.59, 1.39 and 1.26 fold (ADAM9, FBXO11, TRAF3, MAPK1 and MAVS, respectively) less expressed in infected cells 12 h post infection. On the other hand, the pro-inflammatory chemokine C-X-C motif Ligand 2 (CXCL2) had a trending 3.5 fold up-regulation (0.05 ≤ p ≤ 0.1). The Cytochrome C Oxidase assembly subunit 18 (COX18) was the only significantly up-regulated transcript (p ≤ 0.05) with a fold increase of 21.5 times. The significantly down-regulated transcripts include the Decapping Protein 2 (DCP2), Fibronectin Type III Domain Containing 3B (FNDC3B) protein, the chaperone protein Chaperonin Containing TCP1 Subunit 6B (CCT6B) and glutamate transporter Solute Carrier Family 1 Member 2 (SLC1A2) (2.78, 5.47, 17.21 and 39.56 times in infected cells, respectively). The Neurofibromin 1 (NF1), the FYVE, RhoGEF And PH Domain Containing 4 (FGD4), the transcription factor Nuclear Factor I A (NFIA), the Cardiotrophin-Like Cytokine Factor 1 (CLCF1), the Stimulator for Interferon Genes (STING) and the structural component of caveolae invaginations Caveolin 2 (CAV2) were down-regulated (12.57, 11.16, 8.95, 7.96, 7.16 and 6.59 times, respectively) with the same significance (p ≤ 0.01).

In order to evaluate if target regulation could present a higher effect in a later point of the infection, we selected two predicted and published targets for miR-217 and three for miR-576-3p to assess their expression 24 h post infection (Fig 6B). As it was demonstrated that apoptosis is regulated by OROV replication [67], we selected the Mitogen-Activated Protein Kinase 1 (MAPK1) for being a known miR-217 target that regulates apoptosis [68]. Although MAPK1 was not significantly deregulated at 12 h post infection it showed a 2.23 fold down-regulation at 24 h post infection (p ≤ 0.05). The three selected and unpredicted miR-576-3p targets, MAVS, TRAF3 and STING, are known to be important genes in the regulation of IFN-β response in viral infected cells [69]. MAVS and TRAF3 did not presented a significant down-regulation at 12 h post infection (Fig 6A); however, both presented significantly down-regulation at 24 h post infection (2 and 7.4 fold, respectively). STING, the only one of the three selected targets of miR-576-3p that already demonstrated a significant down-regulation at 12 h post infection, showed an even higher down-regulation at 24 h post infection (39 fold down-regulation at 24 h post infection compared to 7.16 at 12 h post infection). The Silent Information Regulator 1 (SIRT1), a histone deacetylase known to be involved in stress-responsive pathways as inflammation [70, 71, 72], was a miR-217 target that did not show significant differential expression relative to uninfected cells 12 h post infection but presented a significant down-regulation at 24 h post infection (3.45 fold down-regulated), what reinforces that different target genes of the same miRNA have different dynamics of regulation. Although not proved as a miRNA target yet, we included DCP2, the only selected miR-217 target already significantly down-regulated at 12 h post infection, because of its relevance as a restriction factor for other bunyavirus [73]. In our model, DCP2 kept a decreasing expression in infected cells, being 20 fold significantly down-regulated at 24 h post infection (p ≤ 0.001).

Overall, we confirmed the modulation of target genes transcription in the opposite direction of its cognate miRNA, showing that miRNA screening is very informative to predict cellular host genes modulated by virus infection.

### IFN-β induction response is attenuated at later stages of infection

**Fig 7:**
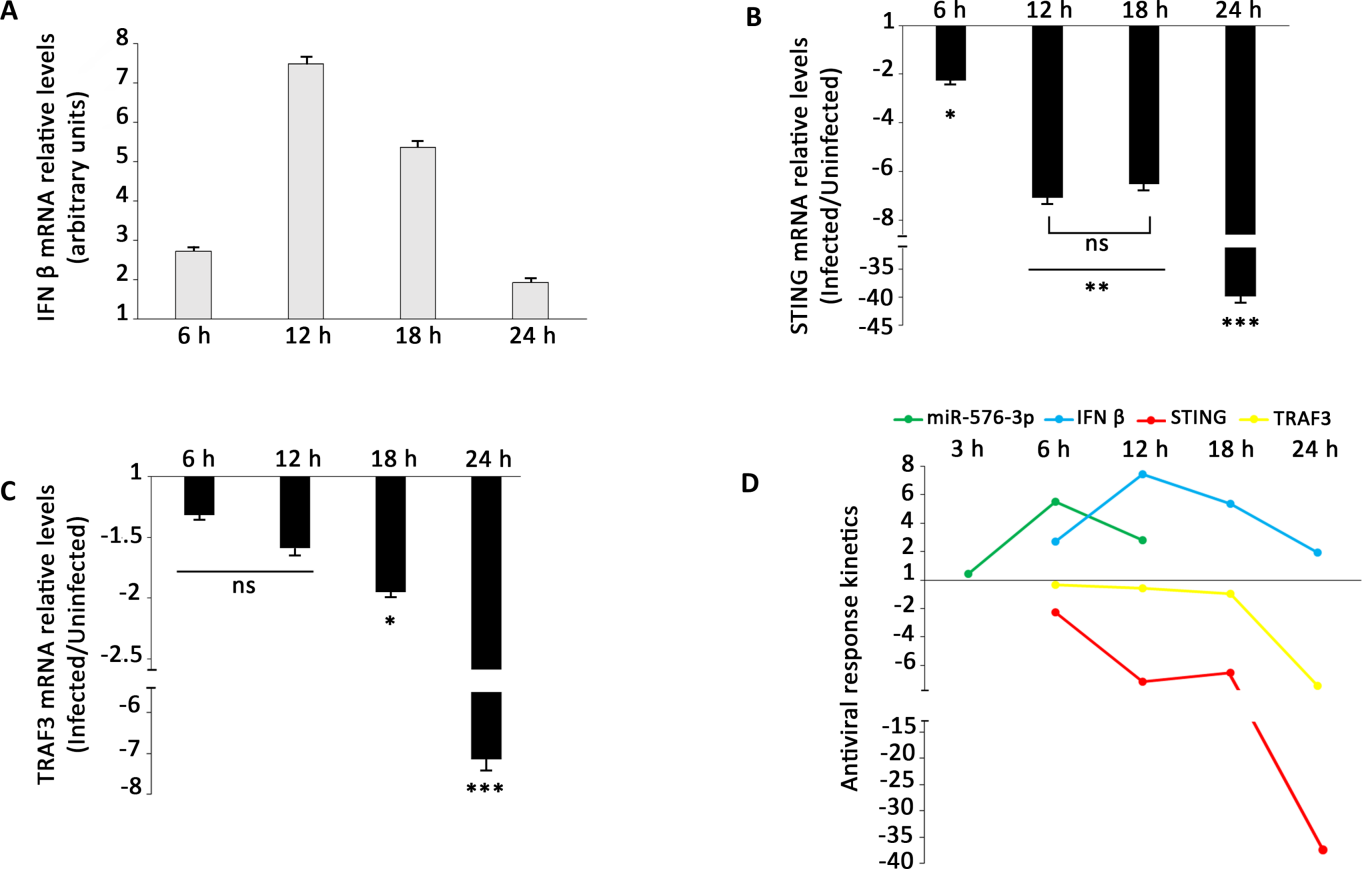
IFN-β antiviral response is attenuated during infection. HuH-7 cells were infected with OROV at MOI 1 and RT-qPCR was done at indicated post-infection time points. (**A**) Normalized expression of IFN-β mRNA during infection. HuH-7 cells were infected with OROV at MOI 1 and IFN-β levels were measured at indicated post-infection time points. Error bars represent SD for four independent infections. Data denotes mean fold change (y-axis) of infected cells relative to uninfected cells for (**B**) STING and (**C**) TRAF3 RNA levels. Gene expression was normalized by endogenous GAPDH levels. Error bars represent SD for four independent samples. ns = non-significant (p ≤ 0.1); * = p ≤ 0.05; ** = p ≤ 0.01; *** = p ≤ 0.001. (**D**) Schematic representation of IFN-β and miR-576-3p interplay in antiviral response during OROV infection. Values are relative to uninfected cells. miR-576-3p, IFN-β, STING and TRAF3 transcripts levels are represented by green, blue, red and yellow lines, respectively.

The type I interferon response is an important canonical innate immunity response mechanism to viral infection. As STING, MAVS and TRAF3 were demonstrated to be key factors in regulation of that response [69], we aimed to quantify the variation in IFN-β transcripts in response to the infection. The IFN-β mRNA levels increased until 12 h post-infection, when it began to drop abruptly, reaching lower levels at 24 post infection (Fig 7A). Those results are consistent with interferon immune response being triggered at early stages of virus replicative cycle. Virus RNA secondary structures are recognized by RIG-I-like receptors (RLR) or toll like receptors members at early stages of virus replication. However, as the infection proceed, the virus induces the miR-576-3p expression promoting the down regulation of its target genes STING and TRAF3 (Fig 7B and 7C)). We hypothesize that OROV try to escape IFN-β response reducing the levels of STING and TRAF3 through miR-576-3p induction (Fig 7D).

### Inhibition of miR-217 and miR-576-3p impairs OROV replication

**Fig 8:**
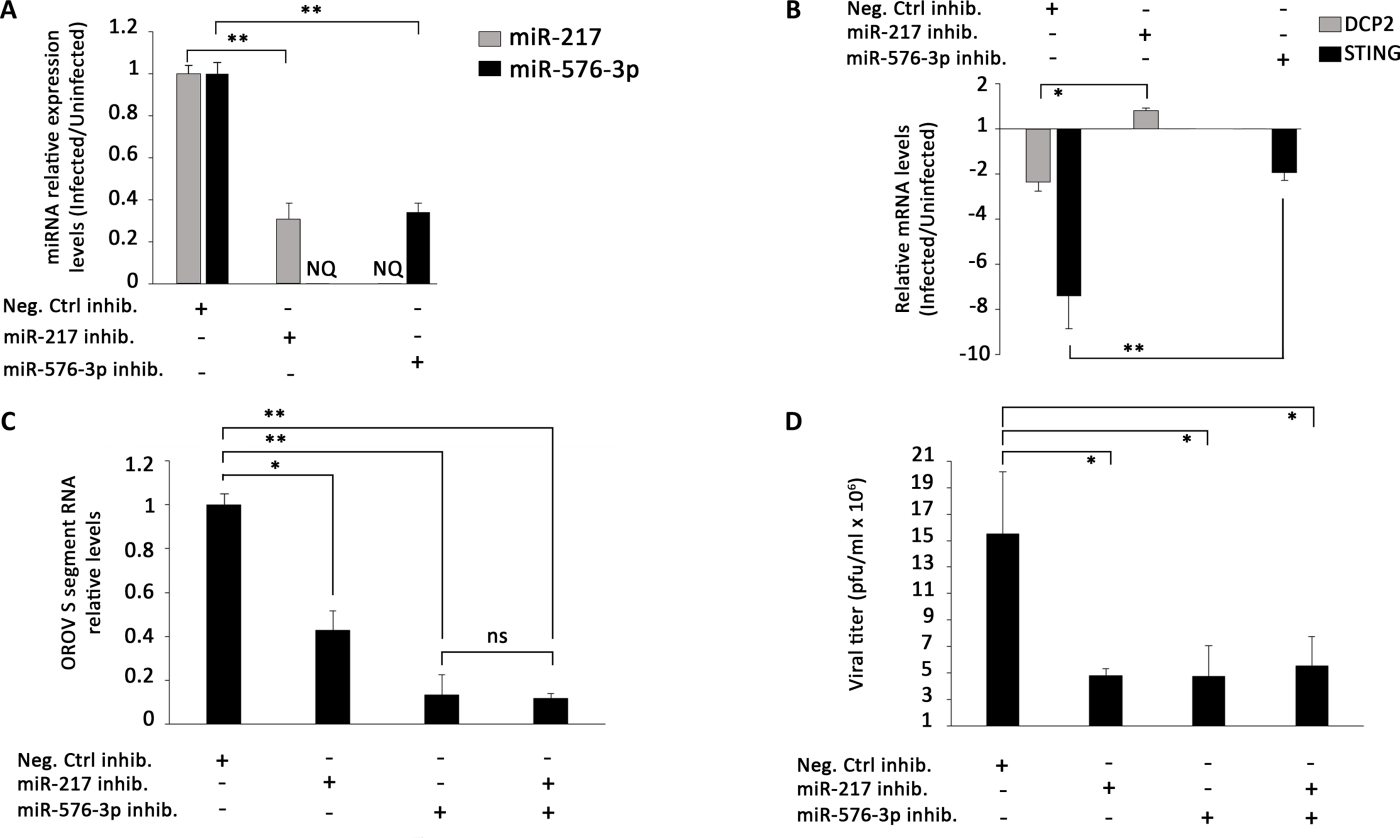
Inhibition of miR-217 and miR-576-3p affects OROV replication. HuH-7 cells were transfected with 75 nM negative control inhibitor, miR-217 inhibitor, miR-576-3p inhibitor or both. 3 h post transfection, cells were infected with OROV at MOI 1 and RT-qPCR was performed to measure miRNA, target genes and OROV RNA levels. (**A**) Fold change of miRNA expression levels 6 h post-infection. MiRNA levels were normalized by U6 levels and infected cells were compared to uninfected cells, both pre-transfected with the respective inhibitor for each condition. Cells transfected with negative inhibitor followed by OROV infection were compared to uninfected cells transfected with the same inhibitor and considered as positive control (set as 1, y-axis) for comparison. Black columns represent miR-217 expression levels and gray columns represent miR-576-3p levels. NQ–not quantified. (**B**) Fold change of target genes RNA levels 18 h post infection. Target genes expression was normalized by GAPDH RNA levels and deregulation was measured relative to the same target in uninfected cells. Black columns represent DCP2 RNA levels and gray columns represent STING RNA levels. (**C**) Intracellular OROV segment S RNA levels 18 h post-infection. Positive control (cells transfected with negative inhibitor and infected with OROV) levels were set as 1 (y-axis) for comparison. (**D**) Viral titration of virus supernatants from experiments performed in **C**. Viruses in the supernatant were quantified by plaque assay 18 h post-infection and plotted as pfu/ml x 10^6^ (y-axis). Error bars represent SD of triplicates for two independent experiments. NS = non-significant; * = p ≤ 0.05; ** = p ≤ 0.01.

In order to assess if miR-217 and miR-576-3p were playing a role in OROV infection, we aimed to evaluate their impact in OROV replication using specific anti-miRNAs (Fig 8).

To accomplish that, HuH-7 cells were transfected with non-human negative control miRNA inhibitor, miR-217 inhibitor, miR-576-3p inhibitor or both miRNA inhibitors and infected with OROV 3h post-transfection. At least 70% of cells were efficiently transfected with negligible cytotoxicity at the concentration tested (S1 Fig). Both miRNA presented a 3-fold decrease in the presence of its respective inhibitor in comparison with negative inhibitor control (Fig 8A). Nonetheless, the predicted target genes for miR-217 and miR-576-3p, DCP2 and STING, respectively, recovered to similar levels to non-infected cells in the presence of miRNA inhibitors 18 h post-infection (Fig 8B). DCP2 RNA levels were slightly above of those in non-infected cells (up to 0.5 fold) whereas STING mRNA levels did not recovered completely but presented a lower decrease compared to the positive control (1.9 and 7.4 fold decrease, respectively).

To further confirm if the miRNA inhibition would influence OROV replication, we measured the intracellular viral RNA levels 18 h post-infection in the presence of miRNA inhibitors (Fig 8C). Inhibition of miR-217 led to a 2.3 fold decrease in viral RNA replication, while inhibition of miR-576-3p led to a 7.7 fold decrease. The highest reduction was observed with inhibition of both miRNAs (8.3 fold), but was not significantly lower than miR-576-3p inhibition alone (Fig 8C). Finally, reduced viral titers confirmed the diminished replication, as a 3-fold decrease was observed in the same time point using miR-217 and miR-576-3p inhibitors (Fig 8D). Altogether, those data demonstrate that inhibition of miR-217 and miR-576-3p is a prospective approach to restrict OROV replication in HuH-7 cells.

## Discussion

In this study, we aimed to identify miRNAs and the target genes regulated in OROV infected hepatocyte cell lines. We demonstrated that miR-217 and miR-576-3p were up-regulated during infection and that their cognate targets were down-regulated. Gene targets related to apoptosis, type I interferon-mediated response and antiviral restriction factors were associated with those miRNAs, suggesting a post transcriptional modulation of those pathways by OROV infection, giving new insights about virus-host interactions.

We initially investigate the susceptibility of human cell lines to the viral infection (Fig 1). Lymphocytes T CD4+ cells (Jurkat) demonstrated low permissiveness to OROV, though being susceptible to infection *in vitro*, as denoted by the 20% of infected cells (Fig 1A). On the other hand, the monocyte cell line THP-1 was not infected in the same conditions. Activation with PMA leads to progressive differentiation of THP-1 into macrophage-like phenotype, as demonstrated elsewhere [58,59,60]. We observed an increase in THP-1 infected population under two different PMA treatment conditions, suggesting a higher susceptibility of those cells as they shift to macrophage phenotype. Indeed, a recent case report detected OROV in peripheral blood mononuclear cells of two patients [74], sustaining the possibility of blood cells playing a role in OROV pathogenesis in humans. In mouse models, however, macrophages only sustained viral replication in immune-compromised individual with deletions in IFN genes [17]. The human hepatocyte cell line HuH-7 was highly permissive to OROV infection, corroborating with previous data that suggest a sustainable liver tropism for OROV [17, 62, 63]. HuH-7 presented a 90% rate of infected cells with no associated cytopathic effect until 18 h post-infection at MOI 1 (Fig 1C), leading us to choose it as our cell model.

We initially found 13 miRNAs differentially expressed in infected cells relative to uninfected cells (Fig 2, Table 1). Some of them were induced while others were modulated upon infection, in agreement with the complexity of miRNA regulation network. MiR-217 was already described in cancer cells involved in tumor migration suppression [75, 76]. Expression kinetics showed a peak at 6 h post-infection for this miRNA (Fig 3A). Regarding the predicted target genes, ten of them were significantly down-regulated 12 h post-infection in RT-qPCR screening (Fig 6A). SLC1A2, also known as Excitatory Amino Acid Transporter 2 (EAAT2) is a glutamate transporter in astrocytes. Lower expression of this transporter was associated with neuropathogenesis outcomes in HIV-1 [77] and Human Herpesvirus 6 (HHV-6) infected cells [78]. In hepatocytes, an increased expression was associated to cholestasis outcome [79]. It is unclear how this transporter could affect OROV infection in hepatocytes, but considering the neurotropism of OROV infection *in vivo*, an investigation of its role in neuropathogenesis should be considered. Another possible cellular factor related to neuropathogenesis of OROV is NF1. The regulation of NF1 by other miRNAs was already demonstrated in neurons and other tissues [80], and is considered a mechanism of fine-tuning in neurological disorders such as neurofibromatosis. The transcription factor NFIA was recently demonstrated to be a novel factor that is negatively regulated by miR-373 [81]. As a consequence, IFN-β response is down-regulated, facilitating Porcine Reproductive and Respiratory Syndrome Virus (PRRSV) replication. The data suggest that both miR-217 and miR-576-3p could act synergistically to inhibit IFN-β antiviral response. CLCF1 is a member of Interleukin-6 (IL-6) family and play a dual role as pro-inflammatory and anti-inflammatory cytokine. FNDC3B has a role in cell migration and invasiveness in hepatocellular carcinoma [82] and glioblastoma cells [83]. In the second case, it was shown that FNDC3B could be down-regulated by miR-129-5p. Likely, FNDC3B could be one of many targets which down-regulation leads to an apoptotic state in OROV infection. The chaperone protein CCT6B as well as its relevance in OROV infection remains elusive. The assembly factor COX18 is a key component for cytochrome oxidase complex works properly [84]. Although its regulation showed an opposite trend, we speculate that this phenomenon could be a collateral effect of the apoptosis state, with cells trying to increase cytochrome oxidase efficiency due to a leaking of cytochrome c to cytoplasm.

We further assessed the expression in a later point of infection (24 h) of three targets of miR-217 during OROV infection: DCP2, MAPK1 and SIRT1 (Fig 6B). DCP2 is a decapping protein involved in mRNA decay. It was recently demonstrated that bunyaviruses compete for the same cellular capped mRNAs that DCP2 targets for degradation in a process known as “cap-snatching” [73]. Bunyaviruses need to snatch capped cellular mRNAs in order to replicate the virus RNA genome; therefore, DCP2 is a direct competitor for bunyaviruses replication. Our results suggested that the down-regulation of DCP2 by miR-217 could explain OROV sustained replication. The kinase MAPK1 and protein SIRT1 both presented a significant lower transcription only 24 h post infection (Fig 6B), when most living cells presumably are in apoptosis process.

The miR-576-3p expression peaked at 6 h post-infection and presented a kinetic similar to miR-217 (Fig 3B). The expression levels at 12 h post-infection were consistent in both quantitative assays (e.g. miRNA array and validation RT-qPCR), corroborating our findings (Table 1 and Fig 3B). At the same time point, two candidate targets, FGD4 and CAV2 were down-regulated, confirming the inverse trend of miR-576-3p (Fig 6A). FGD4 is a protein involved in regulation of actin cytoskeleton and cell migration. Another miRNA, miR-155, was associated to reduced FGD4 levels, resulting in impaired neutrophil migration in myelodysplastic syndromes [85].

CAV2 is a protein component of caveolae structures. A recent study demonstrated that the caveolae and, therefore, its components could act as restriction factor for Tiger Frog Virus (TFV) release in late steps of viral cycle [86] in another hepatocyte cell line, HepG2. As OROV entry is mediated by clathrin-endocytosis [16], we speculate that caveolae could be a restriction site for viral budding/release; therefore down-regulation of a structural component could favors viral release.

MiR-576-3p was recently proposed as a key miRNA in feedback regulation of IFN-β pathway in response to viral infections [69]. Our results regarding down-regulation of STING and TRAF3 corroborated that hypothesis (Fig 6). Moreover, IFN-β transcription regulation correlated with miR-576-3p, STING and TRAF3 transcription dynamics (Fig 7), implying in a temporal feedback mechanism in response to OROV infection, as suggested for other viruses. Based on our data and in conclusions of other group [69], we proposed the following dynamics in antiviral response (depicted in Fig 7D): upon viral entry and uncoating, double-strand viral RNA triggers the IFN-β signaling pathway through STING, MAVS and TRAF3 action, leading Interferon Responsive Factor 3 (IRF3) to activate INF β transcription. Concomitantly, miR-576-3p transcription is also activated by the transcription factor IRF3 and the miRNA accumulation increases until peak 6 h post-infection (Fig 3B). When enough miR-576-3p accumulates in cytoplasm (6 h post-infection) the target mRNA levels, mainly for TRAF3 and STING, begin to fall progressively (Fig 7B and 7C)). At 12 h post-infection, as result of the decrease of STING and TRAF3 mRNA levels, the IFN-β response begins to be relieved, starting a feedback mechanism that leads to a halt in antiviral response and sustaining viral replication.

The miR-576-3p is a primate specific miRNA that was conserved along the evolution, presumably, to avoid tissue damage derived from an excessive inflammatory response due to an infection. Indeed, in mice, OROV infection can be successfully controlled by IFN pathway in immune competent individuals. On the other hand, immune compromised mice (e.g. deleted for genes of IFN pathway) have high mortality rates and fast disease progression with notable liver damage [17]. Our results suggested that, unlike mice, the presence of miR-576-3p in primates and repression of INF-ß rendered them more susceptible to OROV infection.

Furthermore, the inhibition of miR-217 and miR-576-3p partially restricted viral replication, as demonstrated by a decreasing in both viral RNA and titer in the presence of miRNA inhibitors (Fig 8). Those results are in accordance with previous data for miR-576-3p inhibition in other viral infections [69]. We speculate that the restriction is a consequence of a longer sustained innate immune response due to lower suppression of IFN-β pathway signaling cascade in a miRNA inhibition scenery, since both miRNAs might regulate target genes of that pathway.

Finally, as NSs protein has been demonstrated elsewhere to be a candidate viral protein that regulates host innate immune response in other bunyaviruses [87]. A recent study demonstrate that a mutant NSs-deleted OROV induces a strong IFN-α production in opposition to the virus with functional NSs [88]. However, sensitivity to IFN-α treatment was not related to the presence of NSs, as both viruses presented similar sensitivity. It was also demonstrated that OROV is more resistant to IFN-α in comparison to BUNV. Although NSs alone seems to be a candidate viral protein to modulate IFN pathway, we cannot exclude the role of other viral or cellular proteins, as well as viral secondary RNA structures in this conundrum.

We focused our analysis on miR-217 and miR-576-3p given the aforementioned reasons; nonetheless, we cannot exclude the possibility that the other miRNAs identified could be playing a role in OROV infection, as we could validated some of them (Fig 3C). As most of them were star miRNAs and could not be properly investigated by our methodology, a different approach would be necessary to further evaluate if they regulate target genes. Recently, new methodologies to investigate miRNA-mRNAs interactions have been proposed [89, 90] and could be an alternative for future studies. We chose the hepatocyte cell line HuH-7 as our model for an initial, representative study. However, it is also possible that at different time points or using different cell models we could identify different miRNA signatures. It would be interesting to compare the miRNA signature among other permissive cells and using different OROV strains to investigate unique and common miRNA responses to the viral infection. Although the targets validated by RT-qPCR are a good indicative of regulation, those assumptions must be considered with caution, as only RNA down-regulation not necessarily reflects a decrease in protein expression coded by the RNA. Protein quantification (e.g. western blot) would be necessary to assure that the final products of those genes are indeed being regulated. We limited the present study to identify miRNAs and their targets regulated during OROV infection, however, the mechanism by which that regulation occurs remains elusive. A further functional study with expression and knockout of viral proteins could shed a light on the role of viral proteins in this mechanism.

To our knowledge, this is the first study to identify candidate miRNAs that could modulate infection of a member of *Orthobunyavirus* genus, the most representative genus from *Peribunyaviridae* family. Taken together, the data obtained in this study hint at pathways that could impact OROV infection, replication and pathogenesis, and expand the knowledge of the complex interactions in bunyavirus infections.

## Supporting information

S1 Table Oligos used in this study. (DOCX)

S2 Table All 195 predicted target mRNAs found in this study. (DOCX)

S3 Table Selected 95 target mRNAs for RT-qPCR validation and their functions. (DOCX)

S1 Fig miRNA inhibitors transfection efficiency and cytotoxicity. (**A**) Transfection efficiency was assessed using lipofectamine 2000 in two different time points (3 h and 24 h) with 50 nM, 75 nM and 100 nM final concentration of green fluorescent short RNA control (siGLO). Transfected cells (y-axis) were counted (10.000 gated events) using a FITC channel in Accuri C6 flow cytometer. Black columns represent cells 3 h post-transfection and gray columns represent cells 24 h post-transfection. Error bars represent SD of duplicates for two independent experiments. (**B**) Cell viability was assessed using CellTiter Blue 24 h post-transfection with either miRNA inhibitors or both at final concentration of 75 nM. Viability of untransfected cells was set as 1. Error bars represent SD of five replicas for two independent experiments.
(TIF)

## References

[1] Nunes MRT, Martins LC, Rodrigues SG, et al Oropouche Virus Isolation, Southeast Brazil. *Emerg Infect Dis J*. 2005;11(10):1610 10.3201/eid1110.050464 16318707 PMC3366749

[2] Dixon KE, Travassos da Rosa AP, Travassos da Rosa JF, Llewellyn CH. Oropouche virus. II. Epidemiological observations during an epidemic in Santarém, Pará, Brazil in 1975. *Am J Trop Med Hyg*. 1981;30(1):161–164. http://europepmc.org/abstract/MED/7212162. 7212162

[3] Pinheiro FP, Hoch AL, Gomes M de LC, Roberts DR. Oropouche Virus: IV. Laboratory Transmission by Culicoides paraensis. *Am J Trop Med Hyg*. 1981;30 (1):172–176. http://www.ajtmh.org/content/30/1/172.short. 7212164

[4] Vasconcelos HB, Azevedo RSS, Casseb SM, et al Oropouche fever epidemic in Northern Brazil: Epidemiology and molecular characterization of isolates. *J Clin Virol*. 2016;44(2):129–133. 10.1016/j.jcv.2008.11.006 19117799

[5] Baisley KJ, Watts DM, Munstermann LE, Wilson ML. Epidemiology of endemic Oropouche virus transmission in upper Amazonian Peru. *Am J Trop Med Hyg*. 1998;59 (5):710–716. http://www.ajtmh.org/content/59/5/710.abstract. 9840586 10.4269/ajtmh.1998.59.710

[6] Mourão MPG, Bastos MS, Gimaque JBL, et al Oropouche Fever Outbreak, Manaus, Brazil, 2007–2008. *Emerg Infect Dis J*. 2009;15(12):2063 10.3201/eid1512.090917 19961705 PMC3044544

[7] Bastos M de S, Figueiredo LTM, Naveca FG, et al Identification of Oropouche Orthobunyavirus in the Cerebrospinal Fluid of Three Patients in the Amazonas, Brazil. *Am J Trop Med Hyg*. 2012;86 (4):732–735. http://www.ajtmh.org/content/86/4/732.abstract. 10.4269/ajtmh.2012.11-0485 22492162 PMC3403753

[8] Anderson CR, Spence L, Downs WG, Aitken THG. Oropouche Virus: a New Human Disease Agent from Trinidad, West Indies. *Am J Trop Med Hyg*. 1961;10 (4):574–578. http://www.ajtmh.org/content/10/4/574.short. 13683183 10.4269/ajtmh.1961.10.574

[9] Pinheiro FP, Travassos da Rosa APA, Travassos da Rosa JFS, et al Oropouche Virus: I. A Review of Clinical, Epidemiological, and Ecological Findings. *Am J Trop Med Hyg*. 1981;30 (1):149–160. http://www.ajtmh.org/content/30/1/149.short. 6782898

[10] Forshey BM, Guevara C, Laguna-Torres VA, et al Arboviral Etiologies of Acute Febrile Illnesses in Western South America, 2000–2007. *PLoS Negl Trop Dis*. 2010;4(8):e787 10.1371/journal.pntd.0000787 20706628 PMC2919378

[11] Vasconcelos HB, Nunes MRT, Casseb LMN, et al Molecular Epidemiology of Oropouche Virus, Brazil. *Emerg Infect Dis J*. 2011;17(5):800 10.3201/eid1705.101333 21529387 PMC3321770

[12] Watts DM, Lavera V, Callahan J, et al Venezuelan equine encephalitis and Oropouche virus infections among Peruvian army troops in the Amazon region of Peru. *Am J Trop Med Hyg*. 1997;56(6):661–667. http://europepmc.org/abstract/MED/9230800. 9230800 10.4269/ajtmh.1997.56.661

[13] Pinheiro FP, Travassos da Rosa AP, Gomes ML, LeDuc JW, Hoch AL. Transmission of Oropouche virus from man to hamster by the midge Culicoides paraensis. *Science (80-)*. 1982;215(4537):1251 LP—1253. http://science.sciencemag.org/content/215/4537/1251.abstract. 10.1126/science.6800036 6800036

[14] ICTV Virus taxonomy: 2016 release. EC 48, Budapest, Hungary, August 2016. Availabe from: https://data.ictvonline.org/proposals/2016.030a-vM.A.v6.Bunyavirales.pdf.

[15] Elliott RM & Schmaljohn CS. Bunyaviridae In: Knipe DM & Howley PM. Fields Virology. 6.ed. (2013) Philadelphia: Lippincott Williams & Wilkins, 2664.

[16] Santos RIM, Rodrigues AH, Silva ML, et al Oropouche virus entry into HeLa cells involves clathrin and requires endosomal acidification. *Virus Res*. 2008;138(1–2):139–143. 10.1016/j.virusres.2008.08.016 18840482 PMC7114418

[17] Proenca-Modena JL, Sesti-Costa R, Pinto AK, et al Oropouche Virus Infection and Pathogenesis Are Restricted by MAVS, IRF-3, IRF-7, and Type I Interferon Signaling Pathways in Nonmyeloid Cells. Doms RW, ed. *J Virol*. 2015;89(9):4720–4737. 10.1128/JVI.00077-15 25717109 PMC4403474

[18] Lau NC, Lim LP, Weinstein EG, Bartel DP. An Abundant Class of Tiny RNAs with Probable Regulatory Roles in Caenorhabditis elegans. *Science (80-)*. 2001;294(5543):858 LP—862. 10.1126/science.1065062 11679671

[19] Lee RC, Ambros V. An Extensive Class of Small RNAs in Caenorhabditis elegans. *Science (80-)*. 2001;294(5543):862 LP—864. 10.1126/science.1065329 11679672

[20] Lagos-Quintana M, Rauhut R, Lendeckel W, Tuschl T. Identification of Novel Genes Coding for Small Expressed RNAs. *Science (80-)*. 2001;294(5543):853 LP—858. 10.1126/science.1064921 11679670

[21] Ding S-W, Lu R. Virus-derived siRNAs and piRNAs in immunity and pathogenesis. *Curr Opin Virol*. 2011;1(6):533–544. 10.1016/j.coviro.2011.10.028 22180767 PMC3237678

[22] Kapinas K, Delany AM. MicroRNA biogenesis and regulation of bone remodeling. *Arthritis Res Ther*. 2011;13(3):220 10.1186/ar3325 21635717 PMC3218876

[23] Rodriguez A, Griffiths-Jones S, Ashurst JL, Bradley A. Identification of Mammalian microRNA Host Genes and Transcription Units. *Genome Res*. 2004;14 (10a):1902–1910. 10.1101/gr.2722704 15364901 PMC524413

[24] Saini HK, Griffiths-Jones S, Enright AJ. Genomic analysis of human microRNA transcripts. *Proc Natl Acad Sci*. 2007;104 (45):17719–17724. 10.1073/pnas.0703890104 17965236 PMC2077053

[25] Denli AM, Tops BBJ, Plasterk RHA, Ketting RF, Hannon GJ. Processing of primary microRNAs by the Microprocessor complex. *Nature*. 2004;432(7014):231–235. 10.1038/nature03049 15531879

[26] Lee Y, Ahn C, Han J, et al The nuclear RNase III Drosha initiates microRNA processing. *Nature*. 2003;425(6956):415–419. 10.1038/nature01957 14508493

[27] Yi R, Qin Y, Macara IG, Cullen BR. Exportin-5 mediates the nuclear export of pre-microRNAs and short hairpin RNAs. *Genes Dev*. 2003;17 (24):3011–3016. 10.1101/gad.1158803 14681208 PMC305252

[28] Lee Y, Jeon K, Lee J, Kim S, Kim VN. MicroRNA maturation: stepwise processing and subcellular localization. *EMBO J*. 2002;21(17):4663 LP—4670. 12198168 10.1093/emboj/cdf476 PMC126204

[29] Zhang H, Kolb FA, Brondani V, Billy E, Filipowicz W. Human Dicer preferentially cleaves dsRNAs at their termini without a requirement for ATP. *EMBO J*. 2002;21(21):5875 LP—5885. 12411505 10.1093/emboj/cdf582 PMC131079

[30] Hutvágner G, Zamore PD. A microRNA in a Multiple-Turnover RNAi Enzyme Complex. *Science (80-)*. 2002;297(5589):2056 LP—2060. 10.1126/science.1073827 12154197

[31] Hutvagner G. Small RNA asymmetry in RNAi: Function in RISC assembly and gene regulation. *FEBS Lett*. 2005;579(26):5850–5857. 10.1016/j.febslet.2005.08.071 16199039

[32] Valencia-Sanchez MA, Liu J, Hannon GJ, Parker R. Control of translation and mRNA degradation by miRNAs and siRNAs. *Genes Dev*. 2006;20 (5):515–524. 10.1101/gad.1399806 16510870

[33] Beilharz TH, Humphreys DT, Clancy JL, et al microRNA-Mediated Messenger RNA Deadenylation Contributes to Translational Repression in Mammalian Cells. *PLoS One*. 2009;4(8):e6783 10.1371/journal.pone.0006783 19710908 PMC2728509

[34] Humphreys DT, Westman BJ, Martin DIK, Preiss T. MicroRNAs control translation initiation by inhibiting eukaryotic initiation factor 4E/cap and poly(A) tail function. *Proc Natl Acad Sci United States Am*. 2005;102 (47):16961–16966. 10.1073/pnas.0506482102 16287976 PMC1287990

[35] Lewis BP, Burge CB, Bartel DP. Conserved Seed Pairing, Often Flanked by Adenosines, Indicates that Thousands of Human Genes are MicroRNA Targets. *Cell*. 2017;120(1):15–20. 10.1016/j.cell.2004.12.035 15652477

[36] Xie X, Lu J, Kulbokas EJ, et al Systematic discovery of regulatory motifs in human promoters and 3' UTRs by comparison of several mammals. *Nature*. 2005;434(7031):338–345. 10.1038/nature03441 15735639 PMC2923337

[37] Gottwein E, Cullen BR. Viral and cellular microRNAs as determinants of viral pathogenesis and immunity. *Cell Host Microbe*. 2008;3(6):375–387. 10.1016/j.chom.2008.05.002 18541214 PMC3079432

[38] Skalsky RL, Cullen BR. Viruses, microRNAs, and Host Interactions. *Annu Rev Microbiol*. 2010;64:123–141. 10.1146/annurev.micro.112408.134243 20477536 PMC3621958

[39] Henke JI, Goergen D, Zheng J, et al microRNA-122 stimulates translation of hepatitis C virus RNA. *EMBO J*. 2008;27(24):3300–3310. 10.1038/emboj.2008.244 19020517 PMC2586803

[40] Jangra RK, Yi M, Lemon SM. Regulation of Hepatitis C Virus Translation and Infectious Virus Production by the MicroRNA miR-122. *J Virol*. 2010;84(13):6615–6625. 10.1128/JVI.00417-10 20427538 PMC2903297

[41] Jopling CL, Schütz S, Sarnow P. Position-dependent Function for a Tandem MicroRNA miR-122 Binding Site Located in the Hepatitis C Virus RNA Genome. *Cell Host Microbe*. 2008;4(1):77–85. 10.1016/j.chom.2008.05.013 18621012 PMC3519368

[42] Jopling CL, Yi M, Lancaster AM, Lemon SM, Sarnow P. Modulation of Hepatitis C Virus RNA Abundance by a Liver-Specific MicroRNA. *Science (80-)*. 2005;309(5740):1577 LP—1581. 10.1126/science.1113329 16141076

[43] Huang J, Wang F, Argyris E, et al Cellular microRNAs contribute to HIV-1 latency in resting primary CD4+ T lymphocytes. *Nat Med*. 2007;13(10):1241–1247. 10.1038/nm1639 17906637

[44] Nathans R, Chu C, Serquina AK, Lu C-C, Cao H, Rana TM. Cellular microRNA and P-bodies modulate host-HIV-1 interactions. *Mol Cell*. 2009;34(6):696–709. 10.1016/j.molcel.2009.06.003 19560422 PMC2763548

[45] Loveday E-K, Svinti V, Diederich S, Pasick J, Jean F. Temporal- and Strain-Specific Host MicroRNA Molecular Signatures Associated with Swine-Origin H1N1 and Avian-Origin H7N7 Influenza A Virus Infection. *J Virol*. 2012;86(11):6109–6122. 10.1128/JVI.06892-11 22438559 PMC3372180

[46] Shin OS, Kumar M, Yanagihara R, Song J-W. Hantaviruses induce cell type- and viral species-specific host microRNA expression signatures. *Virology*. 2013;446(0):217–224. 10.1016/j.virol.2013.07.036 24074584 PMC4129941

[47] Pepini T, Gorbunova EE, Gavrilovskaya IN, Mackow JE, Mackow ER. Andes Virus Regulation of Cellular MicroRNAs Contributes to Hantavirus-Induced Endothelial Cell Permeability. *J Virol*. 2010;84(22):11929–11936. 10.1128/JVI.01658-10 20844033 PMC2977893

[48] Demir ZC, Bastug A, Bodur H, Ergunay K, Ozkul A. MicroRNA expression profiles in patients with acute Crimean Congo hemorrhagic fever reveal possible adjustments to cellular pathways. *J Med Virol*. 2017;89(3):417–422. 10.1002/jmv.24667 27551771

[49] Meier R, Franceschini A, Horvath P, et al Genome-Wide Small Interfering RNA Screens Reveal VAMP3 as a Novel Host Factor Required for Uukuniemi Virus Late Penetration. Dermody TS, ed. *J Virol*. 2014;88(15):8565–8578. 10.1128/JVI.00388-14 24850728 PMC4135934

[50] R Foundation for Statistical Computing, Vienna, Austria. ISBN 3-900051-07-0, Available from http://www.R-project.org.

[51] Ritz C, Spiess A-N. qpcR: an R package for sigmoidal model selection in quantitative real-time polymerase chain reaction analysis. *Bioinformatics*. 2008;24(13):1549–1551. 10.1093/bioinformatics/btn227 18482995

[52] Vandesompele J, De Preter K, Pattyn F, et al Accurate normalization of real-time quantitative RT-PCR data by geometric averaging of multiple internal control genes. *Genome Biol*. 2002;3(7):research0034.1–research0034.11. http://www.ncbi.nlm.nih.gov/pmc/articles/PMC126239/. 12184808 10.1186/gb-2002-3-7-research0034 PMC126239

[53] Mestdagh P, Van Vlierberghe P, De Weer A, et al A novel and universal method for microRNA RT-qPCR data normalization. *Genome Biol*. 2009;10(6):R64–R64. 10.1186/gb-2009-10-6-r64 19531210 PMC2718498

[54] Basso D, Pesarin F, Salmaso L, Solari A. Permutation Tests for Stochastic Ordering and ANOVA. *Lecture Notes in Statistics*. 2009 Vol 194, 1–35.

[55] Savage RS, Ghahramani Z, Griffin JE, de la Cruz BJ, Wild DL. Discovering transcriptional modules by Bayesian data integration. *Bioinformatics*. 2010;26(12):i158–i167. 10.1093/bioinformatics/btq210 20529901 PMC2881394

[56] Alexa A, Rahnenführer J, Lengauer T. Improved scoring of functional groups from gene expression data by decorrelating GO graph structure. *Bioinformatics*. 2006;22(13):1600–1607. 10.1093/bioinformatics/btl140 16606683

[57] Naveca FG, do Nascimento VA, de Souza VC, Nunes BTD, Rodrigues DSG, Vasconcelos PF da C. Multiplexed reverse transcription real-time polymerase chain reaction for simultaneous detection of Mayaro, Oropouche, and Oropouche-like viruses. Mem Inst Oswaldo Cruz [Internet]. Instituto Oswaldo Cruz, Ministério da Saúde; 2017 7 21;112(7):510–3. Available from: http://www.ncbi.nlm.nih.gov/pmc/articles/PMC5452489/ 10.1590/0074-02760160062 28591313 PMC5452489

[58] Traore K, Trush M a., George M, Spannhake EW, Anderson W, Asseffa A. Signal transduction of phorbol 12-myristate 13-acetate (PMA)-induced growth inhibition of human monocytic leukemia THP-1 cells is reactive oxygen dependent. *Leuk Res*. 2005;29(8):863–879. 10.1016/j.leukres.2004.12.011 15978937

[59] Spano a., Barni S, Sciola L. PMA withdrawal in PMA-treated monocytic THP-1 cells and subsequent retinoic acid stimulation, modulate induction of apoptosis and appearance of dendritic cells. *Cell Prolif*. 2013;46(3):328–347. 10.1111/cpr.12030 23692091 PMC6496477

[60] Shin OS, Yanagihara R, Song J-W. Distinct Innate Immune Responses in Human Macrophages and Endothelial Cells Infected with Shrew-borne Hantaviruses. *Virology*. 2012;434(1):43–49. 10.1016/j.virol.2012.08.004 22944108 PMC3752032

[61] Nayak TK, Mamidi P, Kumar A, et al Regulation of Viral Replication, Apoptosis and Pro-Inflammatory Responses by 17-AAG during Chikungunya Virus Infection in Macrophages. Mehle A, ed. *Viruses*. 2017;9(1):3 10.3390/v9010003 28067803 PMC5294972

[62] Rodrigues AH, Santos RI, Arisi GM, et al Oropouche virus experimental infection in the golden hamster (Mesocrisetus auratus). *Virus Res*. 2011;155(1):35–41. 10.1016/j.virusres.2010.08.009 20727376

[63] Araújo R, Dias LB, Araújo MTF, Pinheiro F, Oliva FP. (1978) Ultrastructural changes in the hamster liver after experimental inoculation with Oropouche arbovirus (type BeAn 19991). Rev. Inst. Med. Trop. São Paulo, v. 20, n.1, p. 45–54. 653220

[64] Santos RI, Almeida MFP, Paula FE, Rodrigues AH, Saranzo AM, Paula AE, et al Experimental infection of suckling mice by subcutaneous inoculation with Oropouche virus. Virus Res [Internet]. 2012;170(1):25–33. Available from: http://www.sciencedirect.com/science/article/pii/S0168170212002559 22877689 10.1016/j.virusres.2012.07.006

[65] Fromm B, Billipp T, Peck LE, et al A Uniform System For The Annotation Of Human microRNA Genes And The Evolution Of The Human microRNAome. *Annu Rev Genet*. 2015;49:213–242. 10.1146/annurev-genet-120213-092023 26473382 PMC4743252

[66] Kozomara A, Griffiths-Jones S. miRBase: annotating high confidence microRNAs using deep sequencing data. *Nucleic Acids Res*. 2014;42(D1):D68–D73. 10.1093/nar/gkt1181. 24275495 PMC3965103

[67] Acrani GO, Gomes R, Proença-Módena JL, da Silva AF, Oliveira Carminati P, Silva ML, et al Apoptosis induced by Oropouche virus infection in HeLa cells is dependent on virus protein expression. Virus Res [Internet]. 2010;149(1):56–63. Available from: http://www.sciencedirect.com/science/article/pii/S0168170210000109 10.1016/j.virusres.2009.12.013 20080135

[68] Zhang N, Lu C, Chen L. miR-217 regulates tumor growth and apoptosis by targeting the MAPK signaling pathway in colorectal cancer. Oncol Lett [Internet]. D.A. Spandidos; 2016 12 13;12(6):4589–97. Available from: http://www.ncbi.nlm.nih.gov/pmc/articles/PMC5228443/ 10.3892/ol.2016.5249 28105166 PMC5228443

[69] Yarbrough ML, Zhang K, Sakthivel R, Forst C V, Posner BA, Barber GN, et al Primate-Specific miR-576-3p Sets Host Defense Signaling Threshold. Nat Commun [Internet]. 2014 9 18;5:4963 Available from: http://www.ncbi.nlm.nih.gov/pmc/articles/PMC4170571/ 10.1038/ncomms5963 25232931 PMC4170571

[70] Zhang H-S, Wu T-C, Sang W-W, Ruan Z. MiR-217 is involved in Tat-induced HIV-1 long terminal repeat (LTR) transactivation by down-regulation of SIRT1. Biochim Biophys Acta—Mol Cell Res [Internet]. 2012 5;1823(5):1017–23. Available from: http://www.sciencedirect.com/science/article/pii/S0167488912000547 10.1016/j.bbamcr.2012.02.014 22406815

[71] Deng S, Zhu S, Wang B, Li X, Liu Y, Qin Q, et al Chronic pancreatitis and pancreatic cancer demonstrate active epithelial-mesenchymal transition profile, regulated by miR-217-SIRT1 pathway. Cancer Lett [Internet]. Elsevier; 2017 5 3;355(2):184–91. Available from: 10.1016/j.canlet.2014.08.007 25172416

[72] Shao Y, Lv C, Wu C, Zhou Y, Wang Q. Mir-217 promotes inflammation and fibrosis in high glucose cultured rat glomerular mesangial cells via Sirt1/HIF-1α signaling pathway. Diabetes Metab Res Rev [Internet]. 2016 9 1;32(6):534–43. Available from: 10.1002/dmrr.2788 26891083

[73] Hopkins KC, McLane LM, Maqbool T, Panda D, Gordesky-Gold B, Cherry S. A genome-wide RNAi screen reveals that mRNA decapping restricts bunyaviral replication by limiting the pools of Dcp2-accessible targets for cap-snatching. Genes Dev [Internet]. Cold Spring Harbor Laboratory Press; 2013 7 1;27(13):1511–25. Available from: http://www.ncbi.nlm.nih.gov/pmc/articles/PMC3713431/ 10.1101/gad.215384.113 23824541 PMC3713431

[74] de Souza Luna LK, Rodrigues AH, Santos RIM, Sesti-Costa R, Criado MF, Martins RB, et al Oropouche virus is detected in peripheral blood leukocytes from patients. J Med Virol [Internet]. 2017 6 1;89(6):1108–11. Available from: 10.1002/jmv.24722 27787907

[75] Zhou W, Song F, Wu Q, Liu R, Wang L, Liu C, et al miR-217 inhibits triple-negative breast cancer cell growth, migration, and invasion through targeting KLF5. PLoS One [Internet]. Public Library of Science; 2017 4 24;12(4):e0176395 Available from: 10.1371/journal.pone.0176395 28437471 PMC5402967

[76] Zhang Li, Li Zhang, Liu Han, et al miR-217 suppresses proliferation, migration, and invasion promoting apoptosis via targeting MTDH in hepatocellular carcinoma [Internet]. Oncology Reports. p. 1772–8. 10.3892/or.2017.5401 28184926

[77] Wang Z, Pekarskaya O, Bencheikh M, Chao W, Gelbard HA, Ghorpade A, et al Reduced expression of glutamate transporter EAAT2 and impaired glutamate transport in human primary astrocytes exposed to HIV-1 or gp120. Virology [Internet]. 2003 7 20;312(1):60–73. Available from: http://www.sciencedirect.com/science/article/pii/S0042682203001818 12890621 10.1016/s0042-6822(03)00181-8

[78] Fotheringham J, Williams EL, Akhyani N, Jacobson S. Human Herpesvirus 6 (HHV-6) Induces Dysregulation of Glutamate Uptake and Transporter Expression in Astrocytes. J Neuroimmune Pharmacol [Internet]. 2008;3(2):105–16. Available from: 10.1007/s11481-007-9084-0 18247129

[79] Najimi M, Stéphenne X, Sempoux C, Sokal E. Regulation of hepatic EAAT-2 glutamate transporter expression in human liver cholestasis. World J Gastroenterol [Internet]. Baishideng Publishing Group Co., Limited; 2014 2 14;20(6):1554–64. Available from: http://www.ncbi.nlm.nih.gov/pmc/articles/PMC3925864/ 10.3748/wjg.v20.i6.1554 24587631 PMC3925864

[80] Paschou M, Doxakis E. Neurofibromin 1 Is a miRNA Target in Neurons. Mott JL, editor. PLoS One [Internet]. San Francisco, USA: Public Library of Science; 2012 10 2;7(10):e46773 Available from: http://www.ncbi.nlm.nih.gov/pmc/articles/PMC3462785/ 10.1371/journal.pone.0046773 23056445 PMC3462785

[81] Chen J, Shi X, Zhang X, Wang A, Wang L, Yang Y, et al MicroRNA 373 Facilitates the Replication of Porcine Reproductive and Respiratory Syndrome Virus by Its Negative Regulation of Type I Interferon Induction. J Virol [Internet]. 2017 2 1;91 (3). Available from: http://jvi.asm.org/content/91/3/e01311-16. 10.1128/JVI.01311-16 PMC5244336 27881653

[82] Lin C-H, Lin Y-W, Chen Y-C, Liao C-C, Jou Y-S, Hsu M-T, et al FNDC3B promotes cell migration and tumor metastasis in hepatocellular carcinoma. Oncotarget [Internet]. Impact Journals LLC; 2016 8 2;7(31):49498–508. Available from: http://www.ncbi.nlm.nih.gov/pmc/articles/PMC5226524/ 10.18632/oncotarget.10374 27385217 PMC5226524

[83] Xu H, Hu Y, Qiu W. Potential mechanisms of microRNA-129-5p in inhibiting cell processes including viability, proliferation, migration and invasiveness of glioblastoma cells U87 through targeting FNDC3B. Biomed Pharmacother [Internet]. 2017 3;87:405–11. Available from: http://www.sciencedirect.com/science/article/pii/S075333221632176X 10.1016/j.biopha.2016.12.100 28068630

[84] Bourens M, Barrientos A. Human mitochondrial cytochrome c oxidase assembly factor COX18 acts transiently as a membrane insertase within the subunit 2 maturation module. J Biol Chem [Internet]. 2017 3 22; Available from: http://www.jbc.org/content/early/2017/03/22/jbc.M117.778514. 10.1074/jbc.M117.778514 PMC5427259 28330871

[85] Cao M, Shikama Y, Kimura H, Noji H, Ikeda K, Ono T, et al Mechanisms of Impaired Neutrophil Migration by MicroRNAs in Myelodysplastic Syndromes. J Immunol [Internet]. 2017 2 21;198(5):1887 LP–1899. Available from: http://www.jimmunol.org/content/198/5/1887.abstract 28130497 10.4049/jimmunol.1600622

[86] He J, Zheng Y-W, Lin Y-F, Mi S, Qin X-W, Weng S-P, et al Caveolae Restrict Tiger Frog Virus Release in HepG2 cells and Caveolae-Associated Proteins Incorporated into Virus Particles. Sci Rep [Internet]. Nature Publishing Group; 2016 2 18;6:21663 Available from: http://www.ncbi.nlm.nih.gov/pmc/articles/PMC4757878/ 10.1038/srep21663 26887868 PMC4757878

[87] Schoen A, Weber F. Orthobunyaviruses and innate immunity induction: alieNSs vs. PredatoRRs. Eur J Cell Biol [Internet]. 2015 7;94(7–9):384–90. Available from: http://www.sciencedirect.com/science/article/pii/S0171933515000606. 10.1016/j.ejcb.2015.06.001 26095300

[88] Tilston-Lunel NL, Acrani GO, Randall RE, Elliott RM. Generation of Recombinant Oropouche Viruses Lacking the Nonstructural Protein NSm or NSs. J Virol [Internet]. 2016 3 1;90 (5):2616–27. Available from: http://jvi.asm.org/content/90/5/2616. 10.1128/JVI.02849-15 PMC4810690 26699638

[89] Martin HC, Wani S, Steptoe AL, Krishnan K, Nones K, Nourbakhsh E, et al Imperfect centered miRNA binding sites are common and can mediate repression of target mRNAs. Genome Biol [Internet]. BioMed Central; 2014 3 14;15(3):R51–R51. Available from: http://www.ncbi.nlm.nih.gov/pmc/articles/PMC4053950/ 10.1186/gb-2014-15-3-r51 24629056 PMC4053950

[90] Tan SM, Kirchner R, Jin J, Hofmann O, McReynolds L, Hide W, et al Sequencing of Captive Target Transcripts Identifies the Network of Regulated Genes and Functions of Primate-Specific miR-522. Cell Rep [Internet]. Elsevier; 2017 5 8;8(4):1225–39. Available from: 10.1016/j.celrep.2014.07.023. 25131211

