# Supplementary material for: MicroRNA and cellular targets profiling reveal miR-217 and miR-576-3p as proviral factors during Oropouche infection
Source: PLoS Negl Trop Dis. 2018 May 29;12(5):e0006508. doi: 10.1371/journal.pntd.0006508 (PMC5993330; doi:10.1371/journal.pntd.0006508)
Supplement: S1 Table — (DOCX) [file pntd.0006508.s001.docx]

| **Target gene** | **Oligo sequence** | **Source** | **Application** |
| --- | --- | --- | --- |
| **hsa-miR-217** | **Forward: 5’ TACTGCATCAGGAACTGATTGG** | **custom designed** | **miRNA qPCR** |
| **hsa-miR-26a-1-3p** | **Forward: 5’ CCTATTCTTGGTTACTTGCACG** | **custom designed** | **miRNA qPCR** |
| **hsa-miR-26a-2-3p** | **Forward: 5’ GGCCTATTCTTGATTACTTGTTTC** | **custom designed** | **miRNA qPCR** |
| **hsa-miR-576-3p** | **Forward: 5’ GGAAGATGTGGAAAAATTGGAATC** | **custom designed** | **miRNA qPCR** |
| **hsa-miR-92a-1-5p** | **Forward: 5’ AGGTTGGGATCGGTTG** | **custom designed** | **miRNA qPCR** |
| **DCP2** | **Forward: 5' GCCATTCCCTTTATCAGACCA**  **Reverse: 5' GGCGGAATTTGGTTCGACT** | **pre-designed*** | **mRNA qPCR** |
| **IFN β** | **Forward: 5' GAAACTGAAGATCTCCTAGCCT**  **Reverse: 5' GCCATCAGTCACTTAAACAGC** | **pre-designed*** | **mRNA qPCR** |
| **MAPK1** | **Forward: 5' CATTCAGCTAACGTTCTGCAC**  **Reverse: 5' GTGATCATGGTCTGGATCTGC** | **pre-designed*** | **mRNA qPCR** |
| **MAVS** | **Forward: 5' CAGAACTGGGCAGTACCC**  **Reverse: 5' AGGAGACAGATGGAGACACA** | **pre-designed*** | **mRNA qPCR** |
| **SIRT1** | **Forward: 5' TTCCTTTGCAACAGCATCTTG**  **Reverse: 5' GTTTCATGATAGCAAGCGGTTC** | **pre-designed*** | **mRNA qPCR** |
| **STING** | **Forward: 5' GCATCAAGGATCGGGTTTACAG**  **Reverse: 5' CTTGACTGTATTGTGACATGGC** | **pre-designed*** | **mRNA qPCR** |
| **TRAF3** | **Forward: 5' GCAGACAGCATGAAGAGCA**  **Reverse: 5' GTCGTGCACACTCAGCAT** | **pre-designed*** | **mRNA qPCR** |
| **GAPDH** | **Forward: 5' ACATCGCTCAGACACCATG**  **Reverse: 5' TGTAGTTGAGGTCAATGAAGGG** | **pre-designed*** | **mRNA qPCR** |
|  |  |  |  |
| **OROV** | **Probe: 5’-/56-FAM/CATTTGAAG/ZEN/CTAGATACGG/3IABkFQ/**  **Forward: 5' TCCGGAGGCAGCATATGTG**  **Reverse: 5' ACAACACCAGCATTGAGCACTT** | **custom designed** | **mRNA qPCR** |
| **GAPDH** | **Probe: 5’-/56-FAM/AAGGTCGGA/ZEN/GTCAACGGATTTGGTC/3IABkFQ/**  **Forward: 5' ACATCGCTCAGACACCATG**  **Reverse: 5' TGTAGTTGAGGTCAATGAAGGG** | **pre-designed*** | **mRNA qPCR** |
| **miR-217 inhibitor** | **5’ U/ZEN/CCAAUCAGUUCCUGAUGCAGU/3ZEN/ 3’** | **pre-designed*** | **miRNA inhibition assay** |
| **miR-576-3p inhibitor** | **5’ G/ZEN/AUUCCAAUUUUUCCACAUCU/3ZEN/ 3’** | **pre-designed*** | **miRNA inhibition assay** |
| **Neg. inhibitor ctrl** | **5’ G/ZEN/CGUAUUAUAGCCGAUUAACG/3ZEN/ 3’** | **pre-designed*** | **miRNA inhibition assay** |
| ***IDT PrimeTime pre-designed primers** | |  |  |
