# Supplementary material for: MicroRNA and cellular targets profiling reveal miR-217 and miR-576-3p as proviral factors during Oropouche infection
Source: PLoS Negl Trop Dis. 2018 May 29;12(5):e0006508. doi: 10.1371/journal.pntd.0006508 (PMC5993330; doi:10.1371/journal.pntd.0006508)
Supplement: S2 Table — (DOCX) [file pntd.0006508.s002.docx]

| **Unigene** | **EntrezID** | **Target Gene Symbol** | **miRNA** |
| --- | --- | --- | --- |
| Hs.717415 | 55331 | ACER3 | hsa-miR-217 |
| Hs.712340 | 55331 | ACER3 | hsa-miR-217 |
| Hs.608049 | 55331 | ACER3 | hsa-miR-217 |
| Hs.606691 | 55331 | ACER3 | hsa-miR-217 |
| Hs.23862 | 55331 | ACER3 | hsa-miR-217 |
| Hs.591756 | 134526 | ACOT12 | hsa-miR-217 |
| Hs.439127 | 134526 | ACOT12 | hsa-miR-217 |
| Hs.618683 | 8754 | ADAM9 | hsa-miR-217 |
| Hs.591852 | 8754 | ADAM9 | hsa-miR-217 |
| Hs.2442 | 8754 | ADAM9 | hsa-miR-217 |
| Hs.721282 | 11216 | AKAP10 | hsa-miR-217 |
| Hs.642676 | 11216 | AKAP10 | hsa-miR-217 |
| Hs.601523 | 11216 | AKAP10 | hsa-miR-217 |
| Hs.733138 | 11217 | AKAP2 | hsa-miR-217 |
| Hs.604711 | 11217 | AKAP2 | hsa-miR-217 |
| Hs.733138 | 445815 | AKAP2 | hsa-miR-217 |
| Hs.604711 | 445815 | AKAP2 | hsa-miR-217 |
| Hs.62180 | 54443 | ANLN | hsa-miR-217 |
| Hs.91791 | 63982 | ANO3 | hsa-miR-217 |
| Hs.644956 | 84168 | ANTXR1 | hsa-miR-217 |
| Hs.625840 | 84168 | ANTXR1 | hsa-miR-217 |
| Hs.610325 | 84168 | ANTXR1 | hsa-miR-217 |
| Hs.165859 | 84168 | ANTXR1 | hsa-miR-217 |
| Hs.729014 | 139322 | APOOL | hsa-miR-217 |
| Hs.711727 | 139322 | APOOL | hsa-miR-217 |
| Hs.657551 | 139322 | APOOL | hsa-miR-217 |
| Hs.648463 | 139322 | APOOL | hsa-miR-217 |
| Hs.512181 | 139322 | APOOL | hsa-miR-217 |
| Hs.84084 | 10513 | APPBP2 | hsa-miR-217 |
| Hs.88252 | 286410 | ATP11C | hsa-miR-217 |
| Hs.733038 | 481 | ATP1B1 | hsa-miR-217 |
| Hs.732568 | 481 | ATP1B1 | hsa-miR-217 |
| Hs.291196 | 481 | ATP1B1 | hsa-miR-217 |
| Hs.709314 | 523 | ATP6V1A | hsa-miR-217 |
| Hs.628196 | 523 | ATP6V1A | hsa-miR-217 |
| Hs.626262 | 523 | ATP6V1A | hsa-miR-217 |
| Hs.477155 | 523 | ATP6V1A | hsa-miR-217 |
| Hs.13261 | 577 | BAI3 | hsa-miR-217 |
| Hs.728997 | 53335 | BCL11A | hsa-miR-217 |
| Hs.628999 | 53335 | BCL11A | hsa-miR-217 |
| Hs.600805 | 53335 | BCL11A | hsa-miR-217 |
| Hs.600137 | 53335 | BCL11A | hsa-miR-217 |
| Hs.370549 | 53335 | BCL11A | hsa-miR-217 |
| Hs.656603 | 145407 | C14orf37 | hsa-miR-217 |
| Hs.535420 | 145407 | C14orf37 | hsa-miR-217 |
| Hs.491941 | 116328 | C8orf34 | hsa-miR-217 |
| Hs.73072 | 10693 | CCT6B | hsa-miR-217 |
| Hs.743222 | 253782 | CERS6 | hsa-miR-217 |
| Hs.604034 | 253782 | CERS6 | hsa-miR-217 |
| Hs.734596 | 1124 | CHN2 | hsa-miR-217 |
| Hs.666750 | 1124 | CHN2 | hsa-miR-217 |
| Hs.653033 | 1124 | CHN2 | hsa-miR-217 |
| Hs.496587 | 91851 | CHRDL1 | hsa-miR-217 |
| Hs.502977 | 23529 | CLCF1 | hsa-miR-217 |
| Hs.611641 | 25932 | CLIC4 | hsa-miR-217 |
| Hs.602330 | 25932 | CLIC4 | hsa-miR-217 |
| Hs.595507 | 25932 | CLIC4 | hsa-miR-217 |
| Hs.592249 | 25932 | CLIC4 | hsa-miR-217 |
| Hs.440544 | 25932 | CLIC4 | hsa-miR-217 |
| Hs.727767 | 9575 | CLOCK | hsa-miR-217 |
| Hs.717498 | 9575 | CLOCK | hsa-miR-217 |
| Hs.671434 | 9575 | CLOCK | hsa-miR-217 |
| Hs.658811 | 9575 | CLOCK | hsa-miR-217 |
| Hs.436975 | 9575 | CLOCK | hsa-miR-217 |
| Hs.707136 | 285521 | COX18 | hsa-miR-217 |
| Hs.356697 | 285521 | COX18 | hsa-miR-217 |
| Hs.607561 | 9586 | CREB5 | hsa-miR-217 |
| Hs.437075 | 9586 | CREB5 | hsa-miR-217 |
| Hs.597717 | 55454 | CSGALNACT2 | hsa-miR-217 |
| Hs.745245 | 8065 | CUL5 | hsa-miR-217 |
| Hs.732754 | 8065 | CUL5 | hsa-miR-217 |
| Hs.679672 | 8065 | CUL5 | hsa-miR-217 |
| Hs.440320 | 8065 | CUL5 | hsa-miR-217 |
| Hs.719458 | 2920 | CXCL2 | hsa-miR-217 |
| Hs.75765 | 2920 | CXCL2 | hsa-miR-217 |
| Hs.129452 | 1602 | DACH1 | hsa-miR-217 |
| Hs.626296 | 117154 | DACH2 | hsa-miR-217 |
| Hs.86603 | 117154 | DACH2 | hsa-miR-217 |
| Hs.714204 | 51339 | DACT1 | hsa-miR-217 |
| Hs.731314 | 167227 | DCP2 | hsa-miR-217 |
| Hs.631156 | 167227 | DCP2 | hsa-miR-217 |
| Hs.627242 | 167227 | DCP2 | hsa-miR-217 |
| Hs.607603 | 167227 | DCP2 | hsa-miR-217 |
| Hs.443875 | 167227 | DCP2 | hsa-miR-217 |
| Hs.40499 | 22943 | DKK1 | hsa-miR-217 |
| Hs.603990 | 9228 | DLGAP2 | hsa-miR-217 |
| Hs.113287 | 9228 | DLGAP2 | hsa-miR-217 |
| Hs.707118 | 80331 | DNAJC5 | hsa-miR-217 |
| Hs.608607 | 80331 | DNAJC5 | hsa-miR-217 |
| Hs.164419 | 80331 | DNAJC5 | hsa-miR-217 |
| Hs.639737 | 1795 | DOCK3 | hsa-miR-217 |
| Hs.476284 | 1795 | DOCK3 | hsa-miR-217 |
| Hs.732208 | 1859 | DYRK1A | hsa-miR-217 |
| Hs.719974 | 1859 | DYRK1A | hsa-miR-217 |
| Hs.368240 | 1859 | DYRK1A | hsa-miR-217 |
| Hs.66185 | 1859 | DYRK1A | hsa-miR-217 |
| Hs.603381 | 79813 | EHMT1 | hsa-miR-217 |
| Hs.495511 | 79813 | EHMT1 | hsa-miR-217 |
| Hs.743897 | 1974 | EIF4A2 | hsa-miR-217 |
| Hs.688848 | 1974 | EIF4A2 | hsa-miR-217 |
| Hs.518475 | 1974 | EIF4A2 | hsa-miR-217 |
| Hs.743897 | 619568 | EIF4A2 | hsa-miR-217 |
| Hs.688848 | 619568 | EIF4A2 | hsa-miR-217 |
| Hs.518475 | 619568 | EIF4A2 | hsa-miR-217 |
| Hs.708428 | 55740 | ENAH | hsa-miR-217 |
| Hs.707594 | 55740 | ENAH | hsa-miR-217 |
| Hs.619045 | 55740 | ENAH | hsa-miR-217 |
| Hs.497893 | 55740 | ENAH | hsa-miR-217 |
| Hs.593576 | 85465 | EPT1 | hsa-miR-217 |
| Hs.189073 | 85465 | EPT1 | hsa-miR-217 |
| Hs.621074 | 114799 | ESCO1 | hsa-miR-217 |
| Hs.464733 | 114799 | ESCO1 | hsa-miR-217 |
| Hs.444082 | 2146 | EZH2 | hsa-miR-217 |
| Hs.624411 | 2201 | FBN2 | hsa-miR-217 |
| Hs.519294 | 2201 | FBN2 | hsa-miR-217 |
| Hs.732171 | 80204 | FBXO11 | hsa-miR-217 |
| Hs.617836 | 80204 | FBXO11 | hsa-miR-217 |
| Hs.605834 | 80204 | FBXO11 | hsa-miR-217 |
| Hs.352677 | 80204 | FBXO11 | hsa-miR-217 |
| Hs.736928 | 2263 | FGFR2 | hsa-miR-217 |
| Hs.622244 | 2263 | FGFR2 | hsa-miR-217 |
| Hs.607420 | 2263 | FGFR2 | hsa-miR-217 |
| Hs.533683 | 2263 | FGFR2 | hsa-miR-217 |
| Hs.593650 | 55137 | FIGN | hsa-miR-217 |
| Hs.118769 | 55137 | FIGN | hsa-miR-217 |
| Hs.660195 | 2335 | FN1 | hsa-miR-217 |
| Hs.629065 | 2335 | FN1 | hsa-miR-217 |
| Hs.626165 | 2335 | FN1 | hsa-miR-217 |
| Hs.203717 | 2335 | FN1 | hsa-miR-217 |
| Hs.520525 | 84624 | FNDC1 | hsa-miR-217 |
| Hs.744888 | 64778 | FNDC3B | hsa-miR-217 |
| Hs.364895 | 64778 | FNDC3B | hsa-miR-217 |
| Hs.159430 | 64778 | FNDC3B | hsa-miR-217 |
| Hs.741222 | 2290 | FOXG1 | hsa-miR-217 |
| Hs.695962 | 2290 | FOXG1 | hsa-miR-217 |
| Hs.632336 | 2290 | FOXG1 | hsa-miR-217 |
| Hs.657373 | 8087 | FXR1 | hsa-miR-217 |
| Hs.592886 | 8087 | FXR1 | hsa-miR-217 |
| Hs.478407 | 8087 | FXR1 | hsa-miR-217 |
| Hs.596854 | 57459 | GATAD2B | hsa-miR-217 |
| Hs.4779 | 57459 | GATAD2B | hsa-miR-217 |
| Hs.660588 | 2665 | GDI2 | hsa-miR-217 |
| Hs.735991 | 284161 | GDPD1 | hsa-miR-217 |
| Hs.631744 | 284161 | GDPD1 | hsa-miR-217 |
| Hs.736672 | 2262 | GPC5 | hsa-miR-217 |
| Hs.655675 | 2262 | GPC5 | hsa-miR-217 |
| Hs.615681 | 2262 | GPC5 | hsa-miR-217 |
| Hs.745169 | 2823 | GPM6A | hsa-miR-217 |
| Hs.727073 | 2823 | GPM6A | hsa-miR-217 |
| Hs.609793 | 2823 | GPM6A | hsa-miR-217 |
| Hs.75819 | 2823 | GPM6A | hsa-miR-217 |
| Hs.605579 | 2898 | GRIK2 | hsa-miR-217 |
| Hs.98262 | 2898 | GRIK2 | hsa-miR-217 |
| Hs.623939 | 2966 | GTF2H2 | hsa-miR-217 |
| Hs.422901 | 2966 | GTF2H2 | hsa-miR-217 |
| Hs.191356 | 2966 | GTF2H2 | hsa-miR-217 |
| Hs.609135 | 2966 | GTF2H2 | hsa-miR-217 |
| Hs.623939 | 728340 | GTF2H2 | hsa-miR-217 |
| Hs.422901 | 728340 | GTF2H2 | hsa-miR-217 |
| Hs.191356 | 728340 | GTF2H2 | hsa-miR-217 |
| Hs.609135 | 728340 | GTF2H2 | hsa-miR-217 |
| Hs.623939 | 728340 | GTF2H2C | hsa-miR-217 |
| Hs.623939 | 730394 | GTF2H2C | hsa-miR-217 |
| Hs.623939 | 728340 | GTF2H2C_2 | hsa-miR-217 |
| Hs.623939 | 730394 | GTF2H2C_2 | hsa-miR-217 |
| Hs.644753 | 9709 | HERPUD1 | hsa-miR-217 |
| Hs.146393 | 9709 | HERPUD1 | hsa-miR-217 |
| Hs.630532 | 59269 | HIVEP3 | hsa-miR-217 |
| Hs.596991 | 59269 | HIVEP3 | hsa-miR-217 |
| Hs.403972 | 59269 | HIVEP3 | hsa-miR-217 |
| Hs.191144 | 6928 | HNF1B | hsa-miR-217 |
| Hs.701288 | 3181 | HNRNPA2B1 | hsa-miR-217 |
| Hs.701179 | 3181 | HNRNPA2B1 | hsa-miR-217 |
| Hs.637950 | 3181 | HNRNPA2B1 | hsa-miR-217 |
| Hs.597480 | 3181 | HNRNPA2B1 | hsa-miR-217 |
| Hs.487774 | 3181 | HNRNPA2B1 | hsa-miR-217 |
| Hs.707173 | 220988 | HNRNPA3 | hsa-miR-217 |
| Hs.622323 | 220988 | HNRNPA3 | hsa-miR-217 |
| Hs.597770 | 220988 | HNRNPA3 | hsa-miR-217 |
| Hs.516539 | 220988 | HNRNPA3 | hsa-miR-217 |
| Hs.712644 | 11100 | HNRNPUL1 | hsa-miR-217 |
| Hs.155218 | 11100 | HNRNPUL1 | hsa-miR-217 |
| Hs.501280 | 5654 | HTRA1 | hsa-miR-217 |
| Hs.445403 | 3376 | IARS | hsa-miR-217 |
| Hs.606009 | 22858 | ICK | hsa-miR-217 |
| Hs.417022 | 22858 | ICK | hsa-miR-217 |
| Hs.17109 | 9452 | ITM2A | hsa-miR-217 |
| Hs.169948 | 3738 | KCNA3 | hsa-miR-217 |
| Hs.608830 | 54793 | KCTD9 | hsa-miR-217 |
| Hs.72071 | 54793 | KCTD9 | hsa-miR-217 |
| Hs.319247 | 9729 | KIAA0408 | hsa-miR-217 |
| Hs.373857 | 11278 | KLF12 | hsa-miR-217 |
| Hs.621693 | 688 | KLF5 | hsa-miR-217 |
| Hs.604315 | 688 | KLF5 | hsa-miR-217 |
| Hs.508234 | 688 | KLF5 | hsa-miR-217 |
| Hs.734869 | 3845 | KRAS | hsa-miR-217 |
| Hs.732231 | 3845 | KRAS | hsa-miR-217 |
| Hs.691064 | 3845 | KRAS | hsa-miR-217 |
| Hs.690627 | 3845 | KRAS | hsa-miR-217 |
| Hs.675166 | 3845 | KRAS | hsa-miR-217 |
| Hs.594741 | 3845 | KRAS | hsa-miR-217 |
| Hs.734126 | 22998 | LIMCH1 | hsa-miR-217 |
| Hs.705704 | 22998 | LIMCH1 | hsa-miR-217 |
| Hs.665200 | 22998 | LIMCH1 | hsa-miR-217 |
| Hs.609815 | 22998 | LIMCH1 | hsa-miR-217 |
| Hs.595535 | 22998 | LIMCH1 | hsa-miR-217 |
| Hs.335163 | 22998 | LIMCH1 | hsa-miR-217 |
| Hs.602486 | 286826 | LIN9 | hsa-miR-217 |
| Hs.602018 | 286826 | LIN9 | hsa-miR-217 |
| Hs.120817 | 286826 | LIN9 | hsa-miR-217 |
| Hs.705567 | 4008 | LMO7 | hsa-miR-217 |
| Hs.666150 | 4008 | LMO7 | hsa-miR-217 |
| Hs.612198 | 4008 | LMO7 | hsa-miR-217 |
| Hs.207631 | 4008 | LMO7 | hsa-miR-217 |
| Hs.652155 | 347731 | LRRTM3 | hsa-miR-217 |
| Hs.735319 | 51747 | LUC7L3 | hsa-miR-217 |
| Hs.655691 | 51747 | LUC7L3 | hsa-miR-217 |
| Hs.597400 | 51747 | LUC7L3 | hsa-miR-217 |
| Hs.130293 | 51747 | LUC7L3 | hsa-miR-217 |
| Hs.134859 | 4094 | MAF | hsa-miR-217 |
| Hs.603308 | 256691 | MAMDC2 | hsa-miR-217 |
| Hs.431850 | 5594 | MAPK1 | hsa-miR-217 |
| Hs.234249 | 9479 | MAPK8IP1 | hsa-miR-217 |
| Hs.657594 | 166968 | MIER3 | hsa-miR-217 |
| Hs.605848 | 166968 | MIER3 | hsa-miR-217 |
| Hs.595273 | 166968 | MIER3 | hsa-miR-217 |
| Hs.110702 | 93380 | MMGT1 | hsa-miR-217 |
| Hs.740565 | 4674 | NAP1L2 | hsa-miR-217 |
| Hs.600944 | 4674 | NAP1L2 | hsa-miR-217 |
| Hs.66180 | 4674 | NAP1L2 | hsa-miR-217 |
| Hs.721732 | 4763 | NF1 | hsa-miR-217 |
| Hs.622370 | 4763 | NF1 | hsa-miR-217 |
| Hs.606512 | 4763 | NF1 | hsa-miR-217 |
| Hs.604118 | 4763 | NF1 | hsa-miR-217 |
| Hs.597889 | 4763 | NF1 | hsa-miR-217 |
| Hs.447837 | 4763 | NF1 | hsa-miR-217 |
| Hs.113577 | 4763 | NF1 | hsa-miR-217 |
| Hs.744898 | 4774 | NFIA | hsa-miR-217 |
| Hs.710546 | 4774 | NFIA | hsa-miR-217 |
| Hs.678749 | 4774 | NFIA | hsa-miR-217 |
| Hs.191911 | 4774 | NFIA | hsa-miR-217 |
| Hs.606655 | 25836 | NIPBL | hsa-miR-217 |
| Hs.481927 | 25836 | NIPBL | hsa-miR-217 |
| Hs.667349 | 4857 | NOVA1 | hsa-miR-217 |
| Hs.609716 | 4857 | NOVA1 | hsa-miR-217 |
| Hs.607686 | 4857 | NOVA1 | hsa-miR-217 |
| Hs.31588 | 4857 | NOVA1 | hsa-miR-217 |
| Hs.744867 | 27020 | NPTN | hsa-miR-217 |
| Hs.744399 | 9818 | NUPL1 | hsa-miR-217 |
| Hs.566317 | 9818 | NUPL1 | hsa-miR-217 |
| Hs.507537 | 9818 | NUPL1 | hsa-miR-217 |
| Hs.604711 | 11217 | PALM2-AKAP2 | hsa-miR-217 |
| Hs.604711 | 445815 | PALM2-AKAP2 | hsa-miR-217 |
| Hs.714086 | 5110 | PCMT1 | hsa-miR-217 |
| Hs.279257 | 5110 | PCMT1 | hsa-miR-217 |
| Hs.646405 | 10015 | PDCD6IP | hsa-miR-217 |
| Hs.611958 | 10015 | PDCD6IP | hsa-miR-217 |
| Hs.595926 | 10015 | PDCD6IP | hsa-miR-217 |
| Hs.475896 | 10015 | PDCD6IP | hsa-miR-217 |
| Hs.530331 | 5160 | PDHA1 | hsa-miR-217 |
| Hs.744901 | 23047 | PDS5B | hsa-miR-217 |
| Hs.706868 | 5428 | POLG | hsa-miR-217 |
| Hs.605212 | 8493 | PPM1D | hsa-miR-217 |
| Hs.286073 | 8493 | PPM1D | hsa-miR-217 |
| Hs.626271 | 151987 | PPP4R2 | hsa-miR-217 |
| Hs.431092 | 151987 | PPP4R2 | hsa-miR-217 |
| Hs.745079 | 11168 | PSIP1 | hsa-miR-217 |
| Hs.575668 | 11168 | PSIP1 | hsa-miR-217 |
| Hs.602564 | 9491 | PSMF1 | hsa-miR-217 |
| Hs.471917 | 9491 | PSMF1 | hsa-miR-217 |
| Hs.706610 | 7803 | PTP4A1 | hsa-miR-217 |
| Hs.607235 | 7803 | PTP4A1 | hsa-miR-217 |
| Hs.593184 | 7803 | PTP4A1 | hsa-miR-217 |
| Hs.227777 | 7803 | PTP4A1 | hsa-miR-217 |
| Hs.745216 | 5814 | PURB | hsa-miR-217 |
| Hs.710064 | 5814 | PURB | hsa-miR-217 |
| Hs.349150 | 5814 | PURB | hsa-miR-217 |
| Hs.444360 | 285282 | RABL3 | hsa-miR-217 |
| Hs.617542 | 54715 | RBFOX1 | hsa-miR-217 |
| Hs.459842 | 54715 | RBFOX1 | hsa-miR-217 |
| Hs.706258 | 9584 | RBM39 | hsa-miR-217 |
| Hs.607178 | 9584 | RBM39 | hsa-miR-217 |
| Hs.599616 | 9584 | RBM39 | hsa-miR-217 |
| Hs.282901 | 9584 | RBM39 | hsa-miR-217 |
| Hs.734318 | 353116 | RILPL1 | hsa-miR-217 |
| Hs.594880 | 353116 | RILPL1 | hsa-miR-217 |
| Hs.472270 | 54453 | RIN2 | hsa-miR-217 |
| Hs.713888 | 23168 | RTF1 | hsa-miR-217 |
| Hs.511096 | 23168 | RTF1 | hsa-miR-217 |
| Hs.717834 | 860 | RUNX2 | hsa-miR-217 |
| Hs.608062 | 860 | RUNX2 | hsa-miR-217 |
| Hs.599884 | 860 | RUNX2 | hsa-miR-217 |
| Hs.535845 | 860 | RUNX2 | hsa-miR-217 |
| Hs.197590 | 860 | RUNX2 | hsa-miR-217 |
| Hs.643333 | 10371 | SEMA3A | hsa-miR-217 |
| Hs.252451 | 10371 | SEMA3A | hsa-miR-217 |
| Hs.601136 | 205564 | SENP5 | hsa-miR-217 |
| Hs.240770 | 205564 | SENP5 | hsa-miR-217 |
| Hs.605544 | 57337 | SENP7 | hsa-miR-217 |
| Hs.529551 | 57337 | SENP7 | hsa-miR-217 |
| Hs.744506 | 6421 | SFPQ | hsa-miR-217 |
| Hs.718162 | 6421 | SFPQ | hsa-miR-217 |
| Hs.355934 | 6421 | SFPQ | hsa-miR-217 |
| Hs.744606 | 9644 | SH3PXD2A | hsa-miR-217 |
| Hs.730611 | 9644 | SH3PXD2A | hsa-miR-217 |
| Hs.724834 | 9644 | SH3PXD2A | hsa-miR-217 |
| Hs.678727 | 9644 | SH3PXD2A | hsa-miR-217 |
| Hs.617660 | 9644 | SH3PXD2A | hsa-miR-217 |
| Hs.627388 | 54414 | SIAE | hsa-miR-217 |
| Hs.369779 | 23411 | SIRT1 | hsa-miR-217 |
| Hs.492348 | 114836 | SLAMF6 | hsa-miR-217 |
| Hs.614269 | 6506 | SLC1A2 | hsa-miR-217 |
| Hs.502338 | 6506 | SLC1A2 | hsa-miR-217 |
| Hs.713936 | 54407 | SLC38A2 | hsa-miR-217 |
| Hs.611839 | 54407 | SLC38A2 | hsa-miR-217 |
| Hs.221847 | 54407 | SLC38A2 | hsa-miR-217 |
| Hs.650158 | 57181 | SLC39A10 | hsa-miR-217 |
| Hs.443874 | 6529 | SLC6A1 | hsa-miR-217 |
| Hs.101745 | 22865 | SLITRK3 | hsa-miR-217 |
| Hs.743897 | 619568 | SNORA4 | hsa-miR-217 |
| Hs.712944 | 11017 | SNRNP27 | hsa-miR-217 |
| Hs.54649 | 11017 | SNRNP27 | hsa-miR-217 |
| Hs.319247 | 9729 | SOGA3 | hsa-miR-217 |
| Hs.614316 | 6664 | SOX11 | hsa-miR-217 |
| Hs.604891 | 6664 | SOX11 | hsa-miR-217 |
| Hs.432638 | 6664 | SOX11 | hsa-miR-217 |
| Hs.707088 | 339745 | SPOPL | hsa-miR-217 |
| Hs.333297 | 339745 | SPOPL | hsa-miR-217 |
| Hs.618565 | 10772 | SRSF10 | hsa-miR-217 |
| Hs.602683 | 10772 | SRSF10 | hsa-miR-217 |
| Hs.3530 | 10772 | SRSF10 | hsa-miR-217 |
| Hs.655499 | 9705 | ST18 | hsa-miR-217 |
| Hs.617730 | 9705 | ST18 | hsa-miR-217 |
| Hs.603186 | 9705 | ST18 | hsa-miR-217 |
| Hs.624663 | 10735 | STAG2 | hsa-miR-217 |
| Hs.496710 | 10735 | STAG2 | hsa-miR-217 |
| Hs.721568 | 55342 | STRBP | hsa-miR-217 |
| Hs.694157 | 55342 | STRBP | hsa-miR-217 |
| Hs.287659 | 55342 | STRBP | hsa-miR-217 |
| Hs.659244 | 3703 | STT3A | hsa-miR-217 |
| Hs.504237 | 3703 | STT3A | hsa-miR-217 |
| Hs.647024 | 6804 | STX1A | hsa-miR-217 |
| Hs.734942 | 6867 | TACC1 | hsa-miR-217 |
| Hs.733343 | 6867 | TACC1 | hsa-miR-217 |
| Hs.642259 | 6867 | TACC1 | hsa-miR-217 |
| Hs.638095 | 6867 | TACC1 | hsa-miR-217 |
| Hs.618514 | 6867 | TACC1 | hsa-miR-217 |
| Hs.501252 | 10579 | TACC2 | hsa-miR-217 |
| Hs.613045 | 64786 | TBC1D15 | hsa-miR-217 |
| Hs.601024 | 64786 | TBC1D15 | hsa-miR-217 |
| Hs.627388 | 84897 | TBRG1 | hsa-miR-217 |
| Hs.707510 | 6996 | TDG | hsa-miR-217 |
| Hs.596784 | 6996 | TDG | hsa-miR-217 |
| Hs.584809 | 6996 | TDG | hsa-miR-217 |
| Hs.173824 | 6996 | TDG | hsa-miR-217 |
| Hs.714158 | 11011 | TLK2 | hsa-miR-217 |
| Hs.611380 | 11011 | TLK2 | hsa-miR-217 |
| Hs.445078 | 11011 | TLK2 | hsa-miR-217 |
| Hs.672344 | 56889 | TM9SF3 | hsa-miR-217 |
| Hs.645062 | 56889 | TM9SF3 | hsa-miR-217 |
| Hs.500674 | 56889 | TM9SF3 | hsa-miR-217 |
| Hs.596503 | 130733 | TMEM178A | hsa-miR-217 |
| Hs.40808 | 130733 | TMEM178A | hsa-miR-217 |
| Hs.743694 | 7114 | TMSB4X | hsa-miR-217 |
| Hs.743543 | 84899 | TMTC4 | hsa-miR-217 |
| Hs.605661 | 57616 | TSHZ3 | hsa-miR-217 |
| Hs.278436 | 57616 | TSHZ3 | hsa-miR-217 |
| Hs.704214 | 5412 | UBL3 | hsa-miR-217 |
| Hs.145575 | 5412 | UBL3 | hsa-miR-217 |
| Hs.672582 | 9958 | USP15 | hsa-miR-217 |
| Hs.601685 | 9958 | USP15 | hsa-miR-217 |
| Hs.597676 | 9958 | USP15 | hsa-miR-217 |
| Hs.708704 | 57687 | VAT1L | hsa-miR-217 |
| Hs.461405 | 57687 | VAT1L | hsa-miR-217 |
| Hs.444212 | 7447 | VSNL1 | hsa-miR-217 |
| Hs.617822 | 23063 | WAPAL | hsa-miR-217 |
| Hs.607158 | 23063 | WAPAL | hsa-miR-217 |
| Hs.599231 | 23063 | WAPAL | hsa-miR-217 |
| Hs.203099 | 23063 | WAPAL | hsa-miR-217 |
| Hs.712681 | 22911 | WDR47 | hsa-miR-217 |
| Hs.570055 | 22911 | WDR47 | hsa-miR-217 |
| Hs.614425 | 55841 | WWC3 | hsa-miR-217 |
| Hs.527524 | 55841 | WWC3 | hsa-miR-217 |
| Hs.745092 | 64328 | XPO4 | hsa-miR-217 |
| Hs.592852 | 64328 | XPO4 | hsa-miR-217 |
| Hs.507452 | 64328 | XPO4 | hsa-miR-217 |
| Hs.708084 | 10138 | YAF2 | hsa-miR-217 |
| Hs.649195 | 10138 | YAF2 | hsa-miR-217 |
| Hs.617142 | 10138 | YAF2 | hsa-miR-217 |
| Hs.707548 | 10730 | YME1L1 | hsa-miR-217 |
| Hs.598552 | 10730 | YME1L1 | hsa-miR-217 |
| Hs.605625 | 91746 | YTHDC1 | hsa-miR-217 |
| Hs.593037 | 91746 | YTHDC1 | hsa-miR-217 |
| Hs.175955 | 91746 | YTHDC1 | hsa-miR-217 |
| Hs.713854 | 7529 | YWHAB | hsa-miR-217 |
| Hs.713768 | 7529 | YWHAB | hsa-miR-217 |
| Hs.643544 | 7529 | YWHAB | hsa-miR-217 |
| Hs.744840 | 7532 | YWHAG | hsa-miR-217 |
| Hs.592470 | 7532 | YWHAG | hsa-miR-217 |
| Hs.636158 | 64145 | ZFYVE20 | hsa-miR-217 |
| Hs.475565 | 64145 | ZFYVE20 | hsa-miR-217 |
| Hs.707820 | 7552 | ZNF711 | hsa-miR-217 |
| Hs.326801 | 7552 | ZNF711 | hsa-miR-217 |
| Hs.604711 | 11217 | AKAP2 | hsa-miR-576-3p |
| Hs.604711 | 445815 | AKAP2 | hsa-miR-576-3p |
| Hs.681718 | 55843 | ARHGAP15 | hsa-miR-576-3p |
| Hs.171011 | 55843 | ARHGAP15 | hsa-miR-576-3p |
| Hs.352412 | 136991 | ASZ1 | hsa-miR-576-3p |
| Hs.13261 | 577 | BAI3 | hsa-miR-576-3p |
| Hs.707759 | 858 | CAV2 | hsa-miR-576-3p |
| Hs.671556 | 858 | CAV2 | hsa-miR-576-3p |
| Hs.603096 | 858 | CAV2 | hsa-miR-576-3p |
| Hs.212332 | 858 | CAV2 | hsa-miR-576-3p |
| Hs.611057 | 26097 | CHTOP | hsa-miR-576-3p |
| Hs.367656 | 1740 | DLG2 | hsa-miR-576-3p |
| Hs.734057 | 85406 | DNAJC14 | hsa-miR-576-3p |
| Hs.605890 | 84288 | EFCAB2 | hsa-miR-576-3p |
| Hs.134857 | 84288 | EFCAB2 | hsa-miR-576-3p |
| Hs.117835 | 121512 | FGD4 | hsa-miR-576-3p |
| Hs.666771 | 10875 | FGL2 | hsa-miR-576-3p |
| Hs.520989 | 10875 | FGL2 | hsa-miR-576-3p |
| Hs.193640 | 54363 | HAO1 | hsa-miR-576-3p |
| Hs.744388 | 8359 | HIST2H4A | hsa-miR-576-3p |
| Hs.597557 | 8359 | HIST2H4A | hsa-miR-576-3p |
| Hs.744388 | 8359 | HIST2H4B | hsa-miR-576-3p |
| Hs.597557 | 8359 | HIST2H4B | hsa-miR-576-3p |
| Hs.731309 | 84376 | HOOK3 | hsa-miR-576-3p |
| Hs.614207 | 84376 | HOOK3 | hsa-miR-576-3p |
| Hs.601106 | 84376 | HOOK3 | hsa-miR-576-3p |
| Hs.594442 | 84376 | HOOK3 | hsa-miR-576-3p |
| Hs.162852 | 84376 | HOOK3 | hsa-miR-576-3p |
| Hs.496984 | 4354 | MPP1 | hsa-miR-576-3p |
| Hs.146079 | 79684 | MSANTD2 | hsa-miR-576-3p |
| Hs.106861 | 64324 | NSD1 | hsa-miR-576-3p |
| Hs.734057 | 29095 | ORMDL2 | hsa-miR-576-3p |
| Hs.604711 | 11217 | PALM2-AKAP2 | hsa-miR-576-3p |
| Hs.604711 | 445815 | PALM2-AKAP2 | hsa-miR-576-3p |
| Hs.129706 | 5078 | PAX4 | hsa-miR-576-3p |
| Hs.718170 | 5529 | PPP2R5E | hsa-miR-576-3p |
| Hs.708243 | 5529 | PPP2R5E | hsa-miR-576-3p |
| Hs.637565 | 5529 | PPP2R5E | hsa-miR-576-3p |
| Hs.596070 | 5529 | PPP2R5E | hsa-miR-576-3p |
| Hs.594418 | 5529 | PPP2R5E | hsa-miR-576-3p |
| Hs.334868 | 5529 | PPP2R5E | hsa-miR-576-3p |
| Hs.618135 | 5860 | QDPR | hsa-miR-576-3p |
| Hs.726367 | 84220 | RGPD5 | hsa-miR-576-3p |
| Hs.720366 | 84220 | RGPD5 | hsa-miR-576-3p |
| Hs.697403 | 84220 | RGPD5 | hsa-miR-576-3p |
| Hs.687703 | 84220 | RGPD5 | hsa-miR-576-3p |
| Hs.666924 | 84220 | RGPD5 | hsa-miR-576-3p |
| Hs.652697 | 84220 | RGPD5 | hsa-miR-576-3p |
| Hs.469630 | 84220 | RGPD5 | hsa-miR-576-3p |
| Hs.726367 | 285190 | RGPD5 | hsa-miR-576-3p |
| Hs.720366 | 285190 | RGPD5 | hsa-miR-576-3p |
| Hs.697403 | 285190 | RGPD5 | hsa-miR-576-3p |
| Hs.687703 | 285190 | RGPD5 | hsa-miR-576-3p |
| Hs.666924 | 285190 | RGPD5 | hsa-miR-576-3p |
| Hs.652697 | 285190 | RGPD5 | hsa-miR-576-3p |
| Hs.469630 | 285190 | RGPD5 | hsa-miR-576-3p |
| Hs.726367 | 84220 | RGPD6 | hsa-miR-576-3p |
| Hs.720366 | 84220 | RGPD6 | hsa-miR-576-3p |
| Hs.697403 | 84220 | RGPD6 | hsa-miR-576-3p |
| Hs.687703 | 84220 | RGPD6 | hsa-miR-576-3p |
| Hs.666924 | 84220 | RGPD6 | hsa-miR-576-3p |
| Hs.652697 | 84220 | RGPD6 | hsa-miR-576-3p |
| Hs.469630 | 84220 | RGPD6 | hsa-miR-576-3p |
| Hs.633703 | 54941 | RNF125 | hsa-miR-576-3p |
| Hs.494648 | 54881 | TEX10 | hsa-miR-576-3p |
| Hs.605162 | 7335 | TMEM189-UBE2V1 | hsa-miR-576-3p |
| Hs.598128 | 7335 | TMEM189-UBE2V1 | hsa-miR-576-3p |
| Hs.605162 | 387522 | TMEM189-UBE2V1 | hsa-miR-576-3p |
| Hs.598128 | 387522 | TMEM189-UBE2V1 | hsa-miR-576-3p |
| Hs.592038 | 24150 | TP53TG3 | hsa-miR-576-3p |
| Hs.592038 | 729264 | TP53TG3 | hsa-miR-576-3p |
| Hs.592038 | 729355 | TP53TG3 | hsa-miR-576-3p |
| Hs.741147 | 24150 | TP53TG3B | hsa-miR-576-3p |
| Hs.592038 | 24150 | TP53TG3B | hsa-miR-576-3p |
| Hs.741147 | 729264 | TP53TG3B | hsa-miR-576-3p |
| Hs.592038 | 729264 | TP53TG3B | hsa-miR-576-3p |
| Hs.741147 | 729355 | TP53TG3B | hsa-miR-576-3p |
| Hs.592038 | 729355 | TP53TG3B | hsa-miR-576-3p |
| Hs.624982 | 24150 | TP53TG3C | hsa-miR-576-3p |
| Hs.574317 | 24150 | TP53TG3C | hsa-miR-576-3p |
| Hs.706716 | 24150 | TP53TG3C | hsa-miR-576-3p |
| Hs.592038 | 24150 | TP53TG3C | hsa-miR-576-3p |
| Hs.624982 | 729264 | TP53TG3C | hsa-miR-576-3p |
| Hs.574317 | 729264 | TP53TG3C | hsa-miR-576-3p |
| Hs.706716 | 729264 | TP53TG3C | hsa-miR-576-3p |
| Hs.592038 | 729264 | TP53TG3C | hsa-miR-576-3p |
| Hs.624982 | 729355 | TP53TG3C | hsa-miR-576-3p |
| Hs.574317 | 729355 | TP53TG3C | hsa-miR-576-3p |
| Hs.706716 | 729355 | TP53TG3C | hsa-miR-576-3p |
| Hs.592038 | 729355 | TP53TG3C | hsa-miR-576-3p |
| Hs.605162 | 7335 | UBE2V1 | hsa-miR-576-3p |
| Hs.598128 | 7335 | UBE2V1 | hsa-miR-576-3p |
| Hs.733678 | 200403 | VWA3B | hsa-miR-576-3p |
| Hs.269977 | 200403 | VWA3B | hsa-miR-576-3p |
