# Supplementary material for: MicroRNA and cellular targets profiling reveal miR-217 and miR-576-3p as proviral factors during Oropouche infection
Source: PLoS Negl Trop Dis. 2018 May 29;12(5):e0006508. doi: 10.1371/journal.pntd.0006508 (PMC5993330; doi:10.1371/journal.pntd.0006508)
Supplement: S3 Table — (DOCX) [file pntd.0006508.s003.docx]

| **Target Gene** | **miRNA** | **Description/Function** |  |  |  |  |  |
| --- | --- | --- | --- | --- | --- | --- | --- |
| ADAM9 | hsa-miR-217 | Membrane protein related to cell-cell and cell-matrix interactions, including fertilization, muscle development, and neurogenesis |  |  |  |  |  |
| AKAP10 | hsa-miR-217 | PKA regulatory subunit ligand |  |  |  |  |  |
| APOOL | hsa-miR-217 | Apolipoprotein O-like protein; unknown function |  |  |  |  |  |
| APPBP2 | hsa-miR-217 | A protein that interacts with microtubules; may participate in cell death regulation |  |  |  |  |  |
| ATP11C | hsa-miR-217 | ATPase component |  |  |  |  |  |
| ATP1B1 | hsa-miR-217 | ATPase beta-chain protein |  |  |  |  |  |
| ATP6V1A | hsa-miR-217 | Vacuolar ATPase component |  |  |  |  |  |
| BCL11A | hsa-miR-217 | Zinc-finger protein; may be involved in lymphoma pathogenesis |  |  |  |  |  |
| CCT6B | hsa-miR-217 | Chaperone protein |  |  |  |  |  |
| CERS6 | hsa-miR-217 | Ceramide synthase; involved in stress induced apoptosis in HeLa cells |  |  |  |  |  |
| CHN2 | hsa-miR-217 | GTP-metabolizing protein; important for cell proliferation and migration |  |  |  |  |  |
| CLCF1 | hsa-miR-217 | JAK-Stat signaling pathway activator; stimulates B cells |  |  |  |  |  |
| CLIC4 | hsa-miR-217 | Chloride channel protein; apoptosis inhibitor |  |  |  |  |  |
| COX18 | hsa-miR-217 | Cytochrome C oxidase assembling component |  |  |  |  |  |
| CREB5 | hsa-miR-217 | CRE-family transactivator protein |  |  |  |  |  |
| CSGALNACT2 | hsa-miR-217 | Protein involved in elongation during chondroitin sulfate synthesis |  |  |  |  |  |
| CUL5 | hsa-miR-217 | E3 ubiquitine ligase |  |  |  |  |  |
| CXCL2 | hsa-miR-217 | Inflammatory cytokine |  |  |  |  |  |
| DACT1 | hsa-miR-217 | Beta-catenin 1 antagonist |  |  |  |  |  |
| DCP2 | hsa-miR-217 | mRNA decapping protein; P-bodies component involved in mRNA decay machinery and competitive inhibitor of bunyaviruses transcription | | | |  |  |
| EHMT1 | hsa-miR-217 | Histone methyltransferase; transcription repressor |  |  |  |  |  |
| EIF4A2 | hsa-miR-217 | Eukaryotic translation initiation factor |  |  |  |  |  |
| FBN2 | hsa-miR-217 | Component of connective tissue microfibrils |  |  |  |  |  |
| FBXO11 | hsa-miR-217 | Ubiquitin-ligase protein complex subunit; regulates apoptosis in melanocytes |  |  |  |  |  |
| FN1 | hsa-miR-217 | Fibronectin 1; acts on cellular adhesion and migration |  |  |  |  |  |
| FNDC3B | hsa-miR-217 | Fibronectin type III domain containing protein |  |  |  |  |  |
| FXR1 | hsa-miR-217 | RNA ligand protein associated to ribosome |  |  |  |  |  |
| GDI2 | hsa-miR-217 | GDP dissociation inhibitor; acts on Rab pathway |  |  |  |  |  |
| GDPD1 | hsa-miR-217 | Hydrolysis of deacylated glycerophospholipids |  |  |  |  |  |
| HERPUD1 | hsa-miR-217 | Ubiquitin domain-containing protein; may acts on protein degradation |  |  |  |  |  |
| HIVEP3 | hsa-miR-217 | Transcription factor; regulates transcription mediates by NF-κB |  |  |  |  |  |
| HNF1B | hsa-miR-217 | Transcription factor in hepatocytes |  |  |  |  |  |
| HNRNPUL1 | hsa-miR-217 | RNA ligand protein; adenovirus oncogenic protein ligand |  |  |  |  |  |
| ITM2A | hsa-miR-217 | Membrane protein involved in osteogenic and chondrogenic differentiation |  |  |  |  |  |
| KCTD9 | hsa-miR-217 | Potassium channel component; role in HBV infection |  |  |  |  |  |
| KLF12 | hsa-miR-217 | Zinc Finger protein; AP-2 transcription factor repressor |  |  |  |  |  |
| LIN9 | hsa-miR-217 | DNA synthesis inhibitor protein |  |  |  |  |  |
| LUC7L3 | hsa-miR-217 | Protein related to splicesome assembly |  |  |  |  |  |
| MAF | hsa-miR-217 | Macrophage activation factor |  |  |  |  |  |
| MAPK1 | hsa-miR-217 | MAP kinase |  |  |  |  |  |
| MAPK8IP1 | hsa-miR-217 | Inhibitor of transcription factors activated by MAPK8 |  |  |  |  |  |
| NF1 | hsa-miR-217 | Neurofibromin 1; may acts as negative regulator of Ras pathway |  |  |  |  |  |
| NFIA | hsa-miR-217 | Transcription factor; involved in HIV-1 integration |  |  |  |  |  |
| NOVA1 | hsa-miR-217 | RNA ligand protein; RNA metabolism regulator in neurons |  |  |  |  |  |
| PCMT1 | hsa-miR-217 | Protein carboxil-methyltransferase; protein repair machinery |  |  |  |  |  |
| PDCD6IP | hsa-miR-217 | Acts on ESCRT endosomal vesicles trafficking pathway |  |  |  |  |  |
| POLG | hsa-miR-217 | Mitochondrial DNA polymerase |  |  |  |  |  |
| PPM1D | hsa-miR-217 | Negative regulator of cellular stress response pathway;apoptosis regulation in stress |  |  |  |  |  |
| PSIP1 | hsa-miR-217 | Transcription factor; involved in HIV-1 integration |  |  |  |  |  |
| PSMF1 | hsa-miR-217 | Proteasome activation inhibitor |  |  |  |  |  |
| PTP4A1 | hsa-miR-217 | Tyrosine phosphatase; acts on cellular proliferation and migration |  |  |  |  |  |
| PURB | hsa-miR-217 | DNA ligand protein; acts on replication and transcription |  |  |  |  |  |
| RABL3 | hsa-miR-217 | Rab-like protein |  |  |  |  |  |
| RBFOX1 | hsa-miR-217 | RNA ligand protein; role in alternative splicing |  |  |  |  |  |
| RBM39 | hsa-miR-217 | RNA ligand protein; involved in transcription regulated by stereoidal hormones |  |  |  |  |  |
| RILPL1 | hsa-miR-217 | Lysosomal protein that interacts with Rab |  |  |  |  |  |
| RIN2 | hsa-miR-217 | Rab5 guanin exchange factor |  |  |  |  |  |
| RTF1 | hsa-miR-217 | May play a role in regulation of transcription elongation process and in chromatin remodelation |  |  |  |  |  |
| SENP5 | hsa-miR-217 | SUMO-specific protease; acts on ubiquitin pathway |  |  |  |  |  |
| SENP7 | hsa-miR-217 | SUMO-specific protease; acts on ubiquitin pathway |  |  |  |  |  |
| SFPQ | hsa-miR-217 | Splicing factor; role in Influenza virus transcription |  |  |  |  |  |
| SIAE | hsa-miR-217 | Sialic acids acetylesterase |  |  |  |  |  |
| SIRT1 | hsa-miR-217 | Protein deacetylase |  |  |  |  |  |
| SLAMF6 | hsa-miR-217 | NK cells activation coreceptor |  |  |  |  |  |
| SLC1A2 | hsa-miR-217 | Glutamate transporter in neuron synapses |  |  |  |  |  |
| SLC39A10 | hsa-miR-217 | Zinc transporter |  |  |  |  |  |
| SNRNP27 | hsa-miR-217 | Splicing pathway protein |  |  |  |  |  |
| SRSF10 | hsa-miR-217 | Splicing pathway protein |  |  |  |  |  |
| STRBP | hsa-miR-217 | DNA ligand protein; interacts with PKR |  |  |  |  |  |
| STT3A | hsa-miR-217 | Oligosaccharyltransferase complex catalytic subunit |  |  |  |  |  |
| STX1A | hsa-miR-217 | Sintaxin 1A; role in exocytic synapses |  |  |  |  |  |
| TBC1D15 | hsa-miR-217 | RAS-like protein; may acts on intracellular trafficking in brain |  |  |  |  |  |
| TMSB4X | hsa-miR-217 | Actin sequestering protein |  |  |  |  |  |
| UBL3 | hsa-miR-217 | Ubiquitin 3-like protein |  |  |  |  |  |
| USP15 | hsa-miR-217 | Ubiquitin-specific protease |  |  |  |  |  |
| XPO4 | hsa-miR-217 | Exportin 4; nucleus-cytoplasm shuttle |  |  |  |  |  |
| YTHDC1 | hsa-miR-217 | RNA ligand protein |  |  |  |  |  |
| ZFYVE20 | hsa-miR-217 | RAB effector; play a role in membrane trafficking |  |  |  |  |  |
| CAV2 | hsa-miR-576-3p | Calveolae component; apoptosis related |  |  |  |  |  |
| DLG2 | hsa-miR-576-3p | Membrane-associated guanylate kinase |  |  |  |  |  |
| DNAJC14 | hsa-miR-576-3p | Hsp 40 homologue; modulates flavivirus replication |  |  |  |  |  |
| FGD4 | hsa-miR-576-3p | Actin cytoskeleton regulator protein; MAPK8 activator |  |  |  |  |  |
| HAO1 | hsa-miR-576-3p | 2-hydroxyacid oxidase located in peroxisome of liver and pancreas cells |  |  |  |  |  |
| HIST2H4A | hsa-miR-576-3p | H4 histone; DNA packaging |  |  |  |  |  |
| HIST2H4B | hsa-miR-576-3p | H4 histone; DNA packaging |  |  |  |  |  |
| HOOK3 | hsa-miR-576-3p | Microtubules tethering protein |  |  |  |  |  |
| MAVS | hsa-miR-576-3p | Induction of IFN-β in response to exogenous RNAs |  |  |  |  |  |
| MPP1 | hsa-miR-576-3p | Membrane-associated guanylate kinase |  |  |  |  |  |
| RGPD5 | hsa-miR-576-3p | RANBP2-like protein; RAN ligand located on cytosol face of nucleus pore |  |  |  |  |  |
| RGPD6 | hsa-miR-576-3p | RANBP2-like protein; RAN ligand located on cytosol face of nucleus pore |  |  |  |  |  |
| RNF125 | hsa-miR-576-3p | E3 ubiquitin ligase; protein degradation |  |  |  |  |  |
| TRAF3 | hsa-miR-576-3p | Induction of IFN-β in response to exogenous RNAs |  |  |  |  |  |
| STING | hsa-miR-576-3p | Induction of IFN-β in response to exogenous DNA and possibly to RNA |  |  |  |  |  |
| UBE2V1 | hsa-miR-576-3p | Ubiquitin-conjugating E2 enzyme variant protein; protein degradation |  |  |  |  |  |
| AKAP2 | hsa-miR-217/hsa-miR-576-3p | PKA regulatory subunit ligand |  |  |  |  |  |
