# Supplementary figures and images for: MicroRNA and cellular targets profiling reveal miR-217 and miR-576-3p as proviral factors during Oropouche infection
Source: PLoS Negl Trop Dis. 2018 May 29;12(5):e0006508. doi: 10.1371/journal.pntd.0006508 (PMC5993330; doi:10.1371/journal.pntd.0006508)

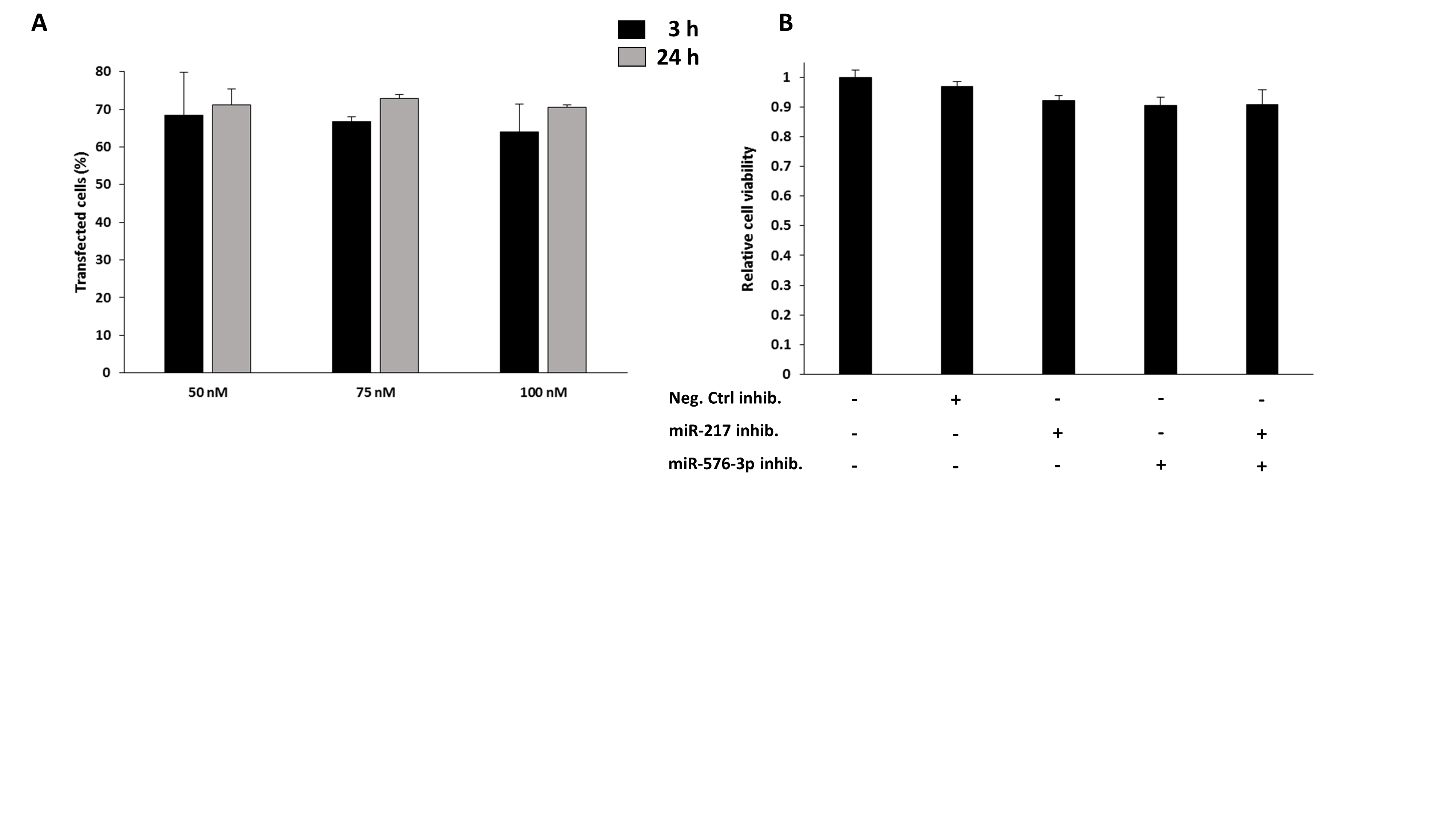

Supplement: S1 Fig — (A) Transfection efficiency was assessed using lipofectamine 2000 in two different time points (3 h and 24 h) with 50 nM, 75 nM and 100 nM final concentration of green fluorescent short RNA control (siGLO). Transfected cells (y-axis) were counted (10.000 gated events) using a FITC channel in Accuri C6 flow cytometer. Black columns represent cells 3 h post-transfection and gray columns represent cells 24 h post-transfection. Error bars represent SD of duplicates for two independent experiments. (B) Cell viability was assessed using CellTiter Blue 24 h post-transfection with either miRNA inhibitors or both at final concentration of 75 nM. Viability of untransfected cells was set as 1. Error bars represent SD of five replicas for two independent experiments. (TIF) [file pntd.0006508.s004.tif]
